# Fungiculture in Termites Is Associated with a Mycolytic Gut Bacterial Community

**DOI:** 10.1128/mSphere.00165-19

**Published:** 2019-05-15

**Authors:** Haofu Hu, Rafael Rodrigues da Costa, Bo Pilgaard, Morten Schiøtt, Lene Lange, Michael Poulsen

**Affiliations:** a Section for Ecology and Evolution, Department of Biology, University of Copenhagen, Copenhagen, Denmark; b Department of Bioengineering, Technical University of Denmark, Lyngby, Denmark; U.S. Department of Energy Joint Genome Institute

**Keywords:** HiSeq, HotPep, carbohydrate-active enzymes, cellulase, chitinase, metagenomics, peptide-based functional predictions

## Abstract

Termites forage on a range of substrates, and it has been suggested that diet shapes the composition and function of termite gut bacterial communities. Through comparative analyses of gut metagenomes in nine termite species with distinct diets, we characterize bacterial community compositions and use peptide-based functional annotation method to determine biomass-degrading enzymes and the bacterial taxa that encode them. We find that fungus-growing termite guts have relatively more fungal cell wall-degrading enzyme genes, while wood-feeding termite gut communities have relatively more plant cell wall-degrading enzyme genes. Interestingly, wood-feeding termite gut bacterial genes code for abundant chitinolytic enzymes, suggesting that fungal biomass within the decaying wood likely contributes to gut bacterial or termite host nutrition. Across diets, the dominant biomass-degrading enzymes are predominantly coded for by the most abundant bacterial taxa, suggesting tight links between diet and gut community composition, with the most marked difference being the communities coding for the mycolytic capacity of the fungus-growing termite gut.

**IMPORTANCE** Understanding functional capacities of gut microbiomes is important to improve our understanding of symbiotic associations. Here, we use peptide-based functional annotation to show that the gut microbiomes of fungus-farming termites code for a wealth of enzymes that likely target the fungal diet the termites eat. Comparisons to other termites showed that fungus-growing termite guts have relatively more fungal cell wall-degrading enzyme genes, whereas wood-feeding termite gut communities have relatively more plant cell wall-degrading enzyme genes. Across termites with different diets, the dominant biomass-degrading enzymes are predominantly coded for by the most abundant bacterial taxa, suggesting tight links between diet and gut community compositions.

## Abstract (precis)

Understanding functional capacities of gut microbiomes is important to improve our understanding of symbiotic associations. Here, we use peptide-based functional annotation to show that the gut microbiomes of fungus-farming termites code for a wealth of enzymes that likely target the fungal diet the termites eat. Comparisons to other termites showed that fungus-growing termite guts have relatively more fungal cell wall-degrading enzyme genes, whereas wood-feeding termite gut communities have relatively more plant cell wall-degrading enzyme genes. Across termites with different diets, the dominant biomass-degrading enzymes are predominantly coded for by the most abundant bacterial taxa, suggesting tight links between diet and gut community compositions.

## INTRODUCTION

Termites are widespread in tropical, subtropical, and warm temperate regions (1) and form a diverse group of more than 3,000 described species in 281 genera and seven families (2–5). They have major impacts on their environments (1), and this success has been attributed to their capacity to use nutritionally imbalanced, recalcitrant food sources, allowing for colonization of otherwise inaccessible niches (6). Different termites forage on distinct substrates, including soil, wood, dung, and fungus (7, 8), decomposed through intricate interactions with complex gut microbial communities (6, 9). In most termites, the main role of gut microbiota is believed to be the digestion of lignocellulose (10, 11), but gut microbes also play key roles in nitrogen fixation (12–14), microbial defense (see, for example, reference 15), and immune regulation (16, 17), which have major importance for the evolutionary history of the symbioses.

Approximately 30 million years ago, the basal higher termite subfamily Macrotermitinae engaged in a mutualistic association with Termitomyces fungi (18, 19) and have a distinct composition of the gut microbiota (20–22). *Termitomyces* decomposes plant material within external fungus gardens (combs) (23, 24), but the gut still remains central in the association because plant substrate is macerated and mixed with asexual *Termitomyces* spores in a first gut passage prior to comb deposition (25). After *Termitomyces* breaks down the plant substrate, the termites ingest mature parts of the comb in a second gut passage (25), where gut microbes may contribute enzymes for final digestion of any remaining plant components (24). This division of labor is consistent with gut bacteria being of importance mainly when the comb material passes through the termite gut in a second passage (cf. references 23, 24, and 26), but recent work has suggested that partial lignin breakdown may also be accomplished during this first gut passage in Odontotermes formosanus (27).

A set of microbes distinct from the gut microbiota of other termites persists in the fungus-growing termite guts, but limited work has examined functional implications of these differences (8, 24). It has been hypothesized that it was associated with the more protein-rich fungal diet (20) and/or to break down chitin and other fungal cell wall components (8, 24, 28). *Termitomyces* domestication exposed fungus-growing termite gut communities to large quantities of fungal cell wall glucans (composed of d-glucose monomers), chitin (glucosamine polymer), and glycoproteins (see, for example, reference 29). Their breakdown requires a combination of carbohydrate-active enzymes (CAZymes; www.cazy.org) (30, 31) and fungus-growing termite gut bacteria indeed encode glycoside hydrolase (GH) families of enzymes that may cleave chitin (GH18, GH19, and GH20), β-glucan (GH55, GH81, and GH128), and α-mannan (GH38, GH76, GH92, GH99, and GH125) (8, 24).

In nature, bacteria are the major chitin degraders and its hydrolysis has been correlated with bacterial abundances in, e.g., soil communities (32). In fungus-growing termites, *Bacteroidetes* and *Firmicutes* bacteria appear to be the main producers of CAZymes putatively producing mycolytic enzymes, i.e., enzymes that lyse the fungal cell wall (8, 24). These studies remained preliminary, however, because they were based on either an unassembled low-coverage metagenome (8) or had limited functional predictions (24). Here, we sequenced the gut metagenome of the fungus-growing termite *Odontotermes* sp. and performed *in silico* analyses to elucidate its fungal and plant cell wall-degrading capacities at deeper functional levels (i.e., to EC numbers when possible), assigned putative enzyme functions to gut community members using peptide-based functional annotation where prediction of function was confirmed by more than one method. To investigate the link between termite diet and gut community composition, we compared our findings to metagenomes from the fungus-growing termite Macrotermes natalensis (24) and seven non-fungus-growing termite species feeding on plant material at different degrees of decomposition: the dung feeder Amitermes wheeleri (33), the two wood feeders Nasutitermes corniger and Microcerotermes parvus, a litter feeder *Cornitermes* sp., the two humus feeders Termes hospes and Neocapritermes taracua, and the soil feeder Cubitermes ugandensis (34). We reveal that the difference in gut community composition is associated with the presence of a mycolytic microbiota, providing insights into digestion and the role of gut communities in the fungus-growing termite symbiosis.

## RESULTS

### Taxonomic composition of fungus-growing termite gut microbiotas.

**FIG 1:**
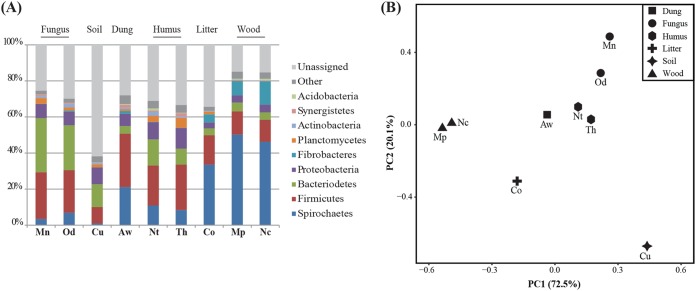
(A) Relative abundances of bacterial phyla each comprising >1% of the microbiota in the guts of termite species with different diets. Termite species are arranged by the degree of plant degradation in the diet. (B) PCA of community similarities of termites with different diets. Mn, *Macrotermes natalensis*; Od, *Odontotermes* sp.; Nc, *Nasutitermes corniger*; Aw, *Amitermes wheeleri*; Mp, *Microcerotermes parvus*; Co, *Cornitermes* sp.; Th, *Termes hospes*; Nt, *Neocapritermes taracua*; Cu, *Cubitermes ugandensis*.

We assigned bacterial taxonomies to metagenome contigs by searching for the closest matches of protein-coding genes on each contig against the NR database in NCBI and compared the relative abundance of the contigs in each group to assess the composition of termite gut microbiotas. *M. natalensis* and *Odontotermes* sp. were distinct from other higher termites primarily being relatively richer in *Bacteroidetes* (Fig. 1A), corroborating previous work (20, 35), and termites in the same feeding group tend to be similar in gut microbiota composition (Fig. 1B; see Table S1 in the supplemental material). Taxonomic compositions at the phylum level were consistent with previous 16S rRNA amplicon surveys of fungus-growing termites (8, 20, 21). *Firmicutes* and *Bacteroidetes* dominated in *Odontotermes* sp. (24 and 25%, respectively) and *M. natalensis* (24 and 30%, respectively) (Fig. 1A; see also Table S1 in the supplemental material), comparable to an average 35 and 32% abundance in 16S-rRNA studies of *Macrotermes subhyalinus* and *Odontotermes* sp. in the Ivory Coast (21) and *Odontotermes yunnanensis* from Southwest China (8). Spirochaetes were relatively abundant in wood-feeding termites (46 to 50%) and *Cornitermes* sp. (32%), and they were low in relative abundance in fungus-growing termites (3 to 6%). At the genus and family levels, we identified the presence of genera that are part of the core gut microbial community in fungus-growing termites (e.g., *Alistipes*, *Treponema*, *Dysgonomonas*, *Desulfovibrio*, *Ruminococcaceae*, and *Lachnospiraceae*) (21). *Alistipes* and *Bacteroides* were most abundant in fungus-growing termites, representing 5 to 8% and 2 to 4% total abundances, respectively, sharply contrasting with other higher termites (on average, 0.1 and 0.5%, respectively) (Table S1). *Treponema* (Spirochaetes) was low in relative abundance in fungus growers and termites feeding on decaying plant material (3% and 6%, respectively), except for the litter feeder *Cornitermes* sp. (32%), while it was the most abundant taxon in wood feeders (42 to 45%) (Table S1). Comparisons to composition estimates from 16S rRNA studies (21, 22, 34) revealed that some genera were underrepresented in the metagenomes. *Alistipes*, for example, were found in much higher relative abundances (12% in average) from classifications using 16S rRNA than protein-coding genes (5 to 8%) (Table S3). Some taxonomic groups classified in 16S rRNA surveys, such as the TG3 phylum (20, 34) were not detected in the metagenomes (Table S1).

10.1128/mSphere.00165-19.1 TABLE S1 Bacterial community composition of metagenome contigs of gut metagenomes of nine termites with different diets at the phylum, class, order, family, and genus levels. Taxonomy classifications with more than 0.1% of the total abundance are shown. Mn, *Macrotermes natalensis*; Od, *Odontotermes* sp.; Nc, *Nasutitermes corniger*; Aw, *Amitermes wheeleri*; Mp, *Microcerotermes parvus*; Co, *Cornitermes* sp.; Th, *Termes hospes*; Nt, *Neocapritermes taracua*; Cu, *Cubitermes ugandensis*. Download Table S1, DOCX file, 0.03 MB. Copyright © 2019 Hu et al. 2019 Hu et al. This content is distributed under the terms of the Creative Commons Attribution 4.0 International license.

### Fungus and plant cell wall-targeting enzymes.

**FIG 2:**
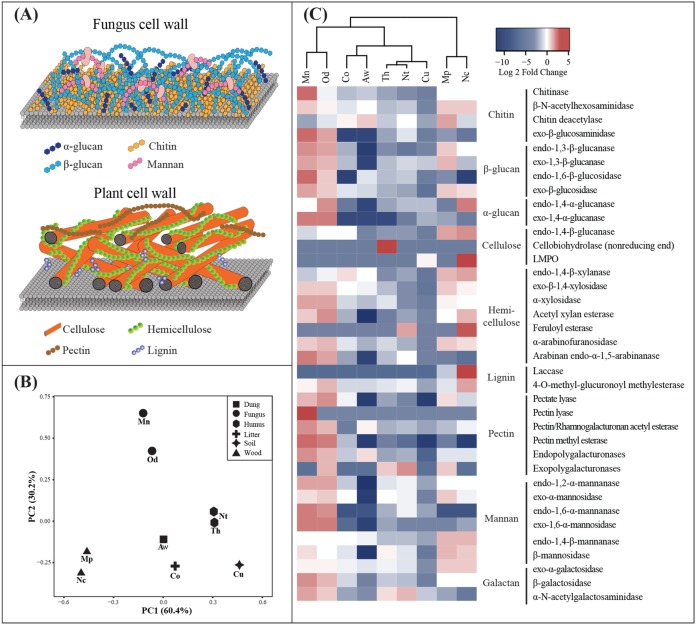
(A) Simplified schematic of fungal (top) and plant (bottom) cell wall structures and targets of enzymes identified in the metagenomes. (B) PCA of relative abundances of carbohydrate-active enzymes in worker gut metagenomes. Shapes represent termite feeding groups. Mn, *Macrotermes natalensis*; Od, *Odontotermes* sp.; Nc, *Nasutitermes corniger*; Aw, *Amitermes wheeleri*; Mp, *Microcerotermes parvus*; Co, *Cornitermes* sp.; Th, *Termes hospes*; Nt, *Neocapritermes taracua*; Cu, *Cubitermes ugandensis*. (C) Higher (red) and lower (blue) relative abundances of enzymes putatively targeting chitin, β-glucan, and α-glucan from the fungal cell wall and cellulose and hemicellulose in nine metagenomes shown as log_2_-transformed fold changes to the average relative abundance across nine species. Mannanases and galactosidases that putatively target plant and fungal cell wall components, respectively, are grouped separately.

To gain insights into the functional capacity for carbohydrates degradation, we first identified carbohydrate-active enzyme (CAZyme) families (30, 31) and classified the genes by their substrate target and thus putative enzyme function by assigning EC numbers using peptide-based functional annotation (36–38) (Table 1; see also Table S2 in the supplemental material). We focus our presentation and comparisons to enzymes, for which the prediction of function was confirmed by more than one method (for details, see Materials and Methods). Principle component analysis (PCA) of glycoside hydrolase (GH) family compositions (Fig. 2B) support that gut microbial enzyme capacities are similar for termites with similar diets. Fungus-growing termite guts were thus systematically different in GH family gene composition to the guts of termites feeding on plant biomass (Fig. 2B; see also Table S2 in the supplemental material), reflecting the differences in cell wall composition of plant and fungus material. Genes encoding GH enzymes that putatively target fungal cell wall components (detailed based on EC numbers in the paragraph below) were more abundant in fungus-growing termites, with the most marked differences being for GH125 (34-fold) and GH92 (14-fold) that likely target α-mannose (Table S2). Genes encoding GH families GH17, GH128, GH55, and GH87 that contain (among others) β-1,3-glucan-targeting enzymes and GH18 and GH19 that include several chitinases showed 2- to 12-fold higher relative abundance in fungus-growing termites (Table S2). In contrast and as expected, many GH family encoding genes primarily targeting plant cell wall components were low in relative abundance in fungus-growing termites but higher in wood-feeding termites (Table S2). Examples include GH94, GH10, and GH5 containing, e.g., cellulases, and GH74, GH120, GH39, GH26, GH10, and GH11 that contain hemi-cellulases, the genes of which were 3- to 5-fold more abundant in wood feeders than in other termites (Table S2).

**TABLE 1:** Relative abundances of enzymes putatively targeting fungal and plant cell wall polysaccharides in the gut metagenomes of nine termites with different diets

EC no.	Enzyme	Substrate	Relative abundance^*a*^								
			Fungus		Soil	Dung	Humus		Litter	Wood	
			Mn	Od	Cu	Aw	Nt	Th	Co	Mp	Nc
3.2.1.14	Chitinase	Chitin	209.59	33.14	3.38	16.89	5.97	8.74	14.70	39.06	43.58
3.2.1.52	β-*N*-Acetylhexosaminidase	Chitin	317.18	208.60	23.51	172.84	84.44	67.93	127.47	362.88	357.39
3.5.1.41	Chitin deacetylase	Chitin	1.79	5.46	2.09	16.89	8.71	3.38	11.30	24.00	4.98
3.2.1.132	Chitosanase	Chitin	0.00	0.00	0.00	0.00	0.00	0.00	0.00	0.00	0.00
3.2.1.165	Exo-β-glucosaminidase	Chitin	34.37	18.35	0.43	0.00	4.56	1.68	0.00	1.35	0.28
3.2.1.39	Endo-1,3-β-glucanase	β-Glucan	45.73	36.28	1.75	0.00	0.68	2.35	4.05	26.47	13.15
3.2.1.58	Exo-1,3-β-glucanase	β-Glucan	21.12	18.12	3.36	0.00	3.92	2.68	2.67	20.37	2.86
3.2.1.75	Endo-1,6-β-glucosidase	β-Glucan	48.53	16.34	0.61	6.14	3.96	3.17	0.00	1.34	0.00
3.2.1.21	Exo-β-glucosidase	β-Glucan	415.89	251.02	4.78	75.52	38.20	47.17	95.79	249.86	207.43
3.2.1.59	Endo-1,3-α-glucosidase	α-Glucan	0.00	0.00	0.00	0.00	0.00	0.00	0.00	0.00	0.00
3.2.1.84	Exo-1,3-α-glucanase	α-Glucan	0.00	0.00	0.00	0.00	0.00	0.00	0.00	0.00	0.00
3.2.1.20	Endo-1,4-α-glucanase	α-Glucan	6.59	21.58	2.27	0.00	2.40	1.67	0.35	0.57	27.73
3.2.1.3	Exo-1,4-α-glucanase	α-Glucan	19.64	16.52	1.25	0.00	0.30	0.00	0.00	1.02	0.31
3.2.1.4	Endo-1,4-β-glucanase	Cellulose	135.62	218.16	16.71	27.63	40.83	41.57	243.08	685.00	793.69
3.2.1.176	Cellobiohydrolase (reducing end)	Cellulose	0.00	0.00	0.00	0.00	0.00	0.00	0.00	0.00	0.00
3.2.1.91	Cellobiohydrolase (nonreducing end)	Cellulose	0.00	0.00	0.00	0.00	0.00	1.42	0.00	0.00	0.00
1.1.99.18	Cellobiose dehydrogenase	Cellulose	0.00	0.00	0.00	0.00	0.00	0.00	0.00	0.00	0.00
1.1.99.29	Pyranose dehydrogenase	Cellulose	0.00	0.00	0.00	0.00	0.00	0.00	0.00	0.00	0.00
LPMO	Lytic polysaccharide monooxygenase	Cellulose	0.00	0.00	0.18	0.00	0.00	0.00	0.00	0.00	1.11
3.2.1.8	Endo-1,4-β-xylanase	Hemicellulose	127.75	204.86	11.58	247.14	57.88	44.17	426.90	591.74	780.19
3.2.1.37	Exo-β-1,4-xylosidase	Hemicellulose	314.18	308.09	5.81	142.76	23.47	58.63	146.37	290.59	199.88
3.2.1.131	Xylan α-1,2-glucuronosidase	Hemicellulose	0.00	0.00	0.00	0.00	0.00	0.00	0.00	0.00	0.00
3.2.1.177	α-Xylosidase	Hemicellulose	167.41	158.69	4.18	19.96	8.43	40.55	30.39	75.39	59.00
3.2.1.151	Xyloglucan endo-β-1,4-glucanase	Hemicellulose	0.00	0.00	0.00	0.00	0.00	0.00	0.00	0.00	0.00
3.1.1.72	Acetyl xylan esterase	Hemicellulose	69.49	114.59	4.62	0.00	9.04	3.26	10.34	35.56	88.05
3.1.1.6	Acetylesterase	Hemicellulose	0.00	0.00	0.00	0.00	0.00	0.00	0.00	0.00	0.00
3.1.1.73	Feruloyl esterase	Hemicellulose	0.00	0.00	0.00	0.00	0.29	0.00	0.00	0.00	0.62
3.2.1.55	α-Arabinofuranosidase	Hemicellulose	184.50	211.25	9.03	27.02	28.15	48.96	57.67	175.31	138.77
3.2.1.99	Arabinan endo-α-1,5-arabinanase	Hemicellulose	31.75	20.05	0.77	0.00	6.81	2.73	1.78	0.60	0.15
1.10.3.2	Laccase	Lignin	0.00	0.00	0.00	0.00	0.00	0.00	0.00	0.25	5.63
3.1.1.-	4-*O*-Methyl-glucuronoyl methylesterase	Lignin	58.92	104.66	11.20	27.63	39.18	35.32	24.60	34.79	131.45
1.11.1.16	Versatile peroxidase	Lignin	0.00	0.00	0.00	0.00	0.00	0.00	0.00	0.00	0.00
4.2.2.2	Pectate lyase	Pectin	36.00	53.84	0.00	0.00	1.97	3.70	11.00	24.54	7.81
4.2.2.10	Pectin lyase	Pectin	0.72	0.00	0.00	0.00	0.00	0.00	0.00	0.00	0.00
3.1.1.-	Pectin acetyl esterase/rhamnogalacturonan acetylesterase	Pectin	106.96	80.44	6.96	39.91	12.42	9.49	2.25	11.76	25.42
3.1.1.11	Pectin methyl esterase	Pectin	72.57	56.74	0.00	0.00	0.28	1.12	5.31	0.94	0.00
3.2.1.15	Endopolygalacturonases	Pectin	92.05	61.05	0.30	38.68	8.83	6.42	11.63	20.46	20.44
3.2.1.67	Exopolygalacturonases	Pectin	0.00	0.92	0.00	0.00	1.22	0.64	0.00	0.71	0.00
3.2.1.174	Rhamnogalacturonan rhamnohydrolase	Pectin	0.00	0.00	0.00	0.00	0.00	0.00	0.00	0.00	0.00
3.2.1.40	α-Rhamnosidase	Pectin	0.00	0.00	0.00	0.00	0.00	0.00	0.00	0.00	0.00
3.2.1.-	Endo-1,2-α-mannanase	Mannan	91.35	92.10	8.77	0.00	29.61	24.99	15.19	34.57	34.26
3.2.1.24	Exo-α-mannosidase	Mannan	20.32	24.65	1.32	0.00	11.59	13.28	14.53	26.42	10.72
3.2.1.101	Endo-1,6-α-mannanase	Mannan	9.73	7.04	0.46	0.00	1.58	0.59	0.00	0.00	0.00
3.2.1.-	Exo-1,6-α-mannosidase	Mannan	39.84	34.46	1.13	0.00	1.07	2.10	0.14	1.14	1.95
3.2.1.78	Endo-1,4-β-mannanase	Mannan	48.81	48.74	11.00	43.29	17.61	15.77	44.69	117.11	109.08
3.2.1.25	β-Mannosidase	Mannan	31.94	21.11	1.16	0.00	12.34	24.07	12.09	45.02	36.08
3.2.1.22	Exo-α-galactosidase	Galactan	116.54	112.55	14.60	59.87	57.90	76.76	77.72	162.74	152.89
3.2.1.23	β-Galactosidase	Galactan	533.54	390.10	13.60	18.73	64.91	71.15	92.29	175.80	181.17
3.2.1.49	α-*N*-Acetylgalactosaminidase	Galactan	138.91	85.11	22.08	7.37	106.47	67.98	10.30	10.49	2.58

a Relative abundances are scaled by multiplication with 10^6^ to improve visualization. Mn, *Macrotermes natalensis*; Od, *Odontotermes* sp.; Nc, *Nasutitermes corniger*; Aw, *Amitermes wheeleri*; Mp, *Microcerotermes parvus*; Co, *Cornitermes* sp.; Th, *Termes hospes*; Nt, *Neocapritermes taracua*; Cu, *Cubitermes ugandensis*.

10.1128/mSphere.00165-19.2 TABLE S2 Relative abundance of glycoside hydrolase families in gut metagenomes of nine species of termites with different diets. Mn, *Macrotermes natalensis*; Od, *Odontotermes* sp.; Nc, *Nasutitermes corniger*; Aw, *Amitermes wheeleri*; Mp, *Microcerotermes parvus*; Co, *Cornitermes* sp.; Th, *Termes hospes*; Nt, *Neocapritermes taracua*; Cu, *Cubitermes ugandensis*. Download Table S2, DOCX file, 0.04 MB. Copyright © 2019 Hu et al. 2019 Hu et al. This content is distributed under the terms of the Creative Commons Attribution 4.0 International license.

Assigning EC numbers to the CAZy genes where possible allowed us to get one step closer to identify putative fungus and plant cell wall substrate targets (Table 1 and Fig. 2C). In fungus-growing termites, the genes of enzymes targeting fungus cell wall polysaccharides were higher in relative abundance, while genes of enzymes targeting plant cell wall components were relatively low (Table 1 and Fig. 2C). For example, the genes encoding chitinase with EC number 3.2.1.14 that endo-hydrolyzes chitin was 5-fold higher in relative abundance in *M. natalensis* (Table 1 and Fig. 2C). Similarly, genes of glucanases targeting 1,3- and 1,6-β-glucan (endo-1,3-β-glucanase, exo-1,3-β-glucanase, and endo-1,6-β-glucosidase) of the fungus cell wall were 2- to 5-fold more abundant in fungus farmers (Table 1 and Fig. 2C). Genes putatively encoding endo-1,2-α-mannanase, exo-α-mannosidase, endo-1,6-α-mannanase, and exo-1,6-α-mannosidase that target α-mannan of the fungus cell wall were more abundant in fungus growers than genes encoding endo-1,4-β-mannanase and β-mannosidase that target the 1,4-β-mannan of hemicellulose in plant cell walls (Fig. 2C). In wood-feeding termites, genes encoding endo-1,4-β-glucanase and endo-1,4-β-xylanase that endo-hydrolyze cellulose and xylan were the most abundant plant cell wall-targeting enzyme genes (Table 1), and they were 2- to 3-fold higher in relative abundance than in other termite guts (Fig. 2C). Genes encoding laccase (EC 1.10.3.2), which plays a role in the cleavage of lignin, were found only in wood-feeding termite guts but in low relative abundance. Genes of several enzymes with exohydrolysis activity, for example, exo-β-glucosidase (EC 3.2.1.21) that hydrolyzes the nonreducing end of glucoses, exo-β-1,4-xylosidase (EC 3.2.1.37) that targets the nonreducing terminal of xylan, and β-*N*-acetylhexosaminidase (EC 3.2.1.52) that hydrolyzes free-end *N*-acetyl-d-hexosamine residues after chitin breakdown were relatively abundant in both fungus-growing and wood-feeding termites (Table 1). Genes encoding galactosidase (3.2.1.22, 3.2.1.23, and 3.2.1.49) that target galactose from the mannan of the fungus cell wall and pectin or hemicellulose components from the plant cell wall were relatively high in abundance in both fungus-growing termites and wood feeding termite guts (Table 1 and Fig. 2C).

### The bacteria encoding the most abundant cell wall-targeting enzymes.

**FIG 3:**
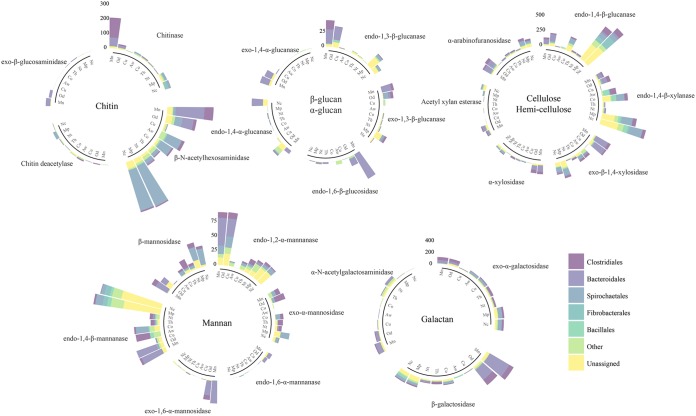
Relative abundances of enzymes putatively targeting chitin, β-glucan, and α-glucan of fungal cell wall and cellulose and hemicellulose encoded by the five most abundant bacterial orders in the nine termite species. Mannanases and galactosidases that target plant and fungal cell wall components, respectively, are shown separately. The relative abundances are scaled by multiplication with 10^6^ to improve visualization. Note that only bar length reflects relative abundance, not bar width or area. Mn, *Macrotermes natalensis*; Od, *Odontotermes* sp.; Nc, *Nasutitermes corniger*; Aw, *Amitermes wheeleri*; Mp, *Microcerotermes parvus*; Co, *Cornitermes* sp.; Th, *Termes hospes*; Nt, *Neocapritermes taracua*; Cu, *Cubitermes ugandensis*.

To gain further insight into the functional contribution of gut microbiota members to carbohydrate degradation, we grouped enzymes targeting polysaccharides from fungus and plant cell wall components by their taxonomy (Fig. 3). *Clostridiales* and *Bacteroidales* contributed most of the cell wall-degrading enzyme genes in fungus-growing termites (83% in *M. natalensis* and 68% in *Odontotermes* sp.). The two orders may, however, differ in what enzymes they contribute. The majority of the endo-1,2-α-mannanases, endo-1,6-α-mannanases, and exo-1,6-α-mannosidases (60%) that target α-mannan of the fungus cell wall were coded for by members of *Bacteroidales*, whereas the exo-α-mannosidases were mainly contributed by members of *Clostridiales* (Fig. 3). Members from both orders contribute genes for endo-1,3-β-glucanase and exo-1,3-β-glucanase that target β-glucan in the fungus cell wall, but only *Bacteroidales* contribute endo-1,6-β-glucosidase.

In wood feeders, genes encoding enzymes for cellulose and hemicellulose cleavage were coded for by all abundant members of the gut microbiotas (Fig. 3). *Spirochaetales* contributed 32% of the cell wall-degrading enzyme genes (Fig. 3), but notably, a large proportion of these genes (36%) could not be assigned to bacterial orders. Genes putatively encoding chitinases and β-N-acetylhexosaminidases for chitin degradation were contributed by both Clostridiales and Bacteroidales and, to a lesser extent, *Spirochaetales* in wood feeders. In termite species that were neither fungus nor wood feeding, most of the fungus cell wall-degrading enzyme genes were also contributed by *Clostridiales*, *Bacteroidales*, and *Spirochaetales* (17 to 92%), but the relative abundances of these enzyme genes were far lower than those observed in fungus and wood feeders (Table 1 and Fig. 3).

## DISCUSSION

Diet is a major driver of taxonomic and functional composition of gut microbial communities (39–41). Our characterization of cell wall degrading enzyme from higher termites confirms that the functional profiles of gut microbial communities are tightly linked with termite diet, which is consistent with previous work that has focused on community compositions (21, 22, 35), and preliminary functional associations with microbiota structure in the Macrotermitinae (8, 24). Fungus-growing termite diets consist of fungal hyphae and plant material that is partly degraded by the symbiotic fungus (22). The gut microbiota thus not only functionally complements final plant biomass decomposition by providing oligosaccharide-targeting enzymes (8, 24) but also provides key enzymes for the digestion of fungal biomass. The mycolytic potential of the gut microbiota of the South African *Odontotermes* sp. and *M. natalensis* (this study) was comparable to *O. yunnanensis* from Southwest China (8), suggesting conserved functions across space and time and highlighting the robust link between taxonomy and function of the intimate interactions between gut community members and termite hosts.

Fungus-growing termite gut mycolytic enzymes were primarily coded for by *Clostridiales* and *Bacteroidales*, which also dominate the core microbiota of the termite subfamily (20, 21, 35, 43, 44). The ancestor of the Macrotermitinae likely had a bacterial gut microbiota similar to those of lower termites (but without protists) (6, 9). Fungiculture exposed the gut microbiota to larger amounts of fungal biomass than the ancestral lignocellulolytic diet, likely resulting in gut microbiotas that became more similar to those observed in extant cockroaches (20, 21). This is likely a product of several factors. First, bacterial strains present in the Macrotermitinae ancestor that could utilize a fungal diet were likely selected for and consequently increased in relative abundance (e.g., *Alistipes*, *Dysgonomonas*, and members of the *Ruminococcaceae*) (20, 21). Many mycolytic microbes were conceivably already present in termite guts prior to the origin of fungiculture, since the ancestral termites fed on partially degraded plant substrates containing fungal biomass. Second, bacteria that do not contribute to the breakdown of fungal material in the Macrotermitinae ancestor were likely outcompeted/selected against in the fungal-material-rich environment. Third, novel lineages adopted from other termites or the environment were likely coopted when fungiculture evolved. This is consistent with recent work demonstrating rampant horizontal transmission of gut bacterial lineages associated with termites (45). Collectively, this led to a gut microbiota with relatively more mycolytic enzymes (i.e., α-mannanases, β-1,3/1,6-glucanases, and chitinases) and relatively fewer lignocellulolytic enzymes (i.e., cellulase and hemicellulose).

In sharp contrast to the fungus growers, lignocellulose-degrading enzymes dominated the gut microbiota of wood-feeding termites (*N. corniger* and *M. parvus*), as expected from the requirement for the breakdown of recalcitrant plant components, which in fungus farmers is handled by *Termitomyces* (24; although lignin cleavage may be initiated during the first gut passage [27]). Interestingly, however, the relatively high abundance of chitinolytic enzymes of *Spirochaetales* origin in wood-feeding termites suggests that the decaying wood these species feed on harbors fungal biomass that gut bacteria, or the termite host, likely utilize. However, fungal cell wall-targeting enzymes such as β-1,3-glucanase may also serve to protect against fungal infections (cf. reference 46). Experimental work targeting the expression of these enzymes in the presence of fungal pathogens, ideally combined with a varying fungal biomass diet content, have the potential to shed light on their relative defensive and dietary roles.

An important caveat of our study is that we were limited by comparisons of bacterial abundances and encoded enzymes between whole-gut DNA extractions and metagenomes in the two fungus-growing termites with P3 compartment of wood- and litter-feeding termites and the lumen metagenome of the dung feeder. This may have biased our comparative analyses somewhat, but we believe that at least the P3 comparison to whole guts is reliable for the following reasons. First, the P3 compartment is expected to contain the vast majority of microbial cells of termite guts (e.g., >97% in the wood feeder *N. corniger* [47]). Consequently, even if relative abundances may differ across gut sections (cf. reference 34), the overall community structure and functions should be primarily driven by P3-residing bacteria. Nevertheless, the contribution of enzyme genes from some member such as *Clostridiales*, which is consistently more abundant in the P1 compartment (48), may have been underestimated when only the P3 region was sequenced. Second, comparisons of community structure between whole gut 16S rRNA from fungus-growing termite (21) with P3 community analyses (20) found nearly identical community structure (see Fig. 4 in reference 26). More importantly, although community composition of the gut fluid of *N. corniger* (33) largely resemble hindgut and P3 compartment analyses (20, 34, 47, 48), we advocate that the comparisons to the lumen metagenome of *A. wheeleri* is taken with a grain of salt for two main reasons. First, lumen and gut wall-associated microbiomes may differ substantially (49, 50). Second, although we normalize our comparisons by metagenome size, the low coverage of *A. wheeleri* most likely undersamples the true composition of the metagenome, thus probably precluding the identification of low-abundance bacteria and enzyme genes (Table 1). Third, although the impact is expected to be minor (cf. reference 42), discrepancies could be impacted by sequencing protocols (454 versus Illumina) due to potential sequencing biases associated with GC content, sequencing errors, and differences in read length.

The shotgun metagenomics data showed distinct compositional profiles of cell wall-degrading enzyme genes in termite guts consistent with expected gut community functions. However, without experimental data of the bacteria densities and complete bacteria genomes, our comparisons cannot reflect enzyme quantities and *in situ* activities (cf. reference 51). A large fraction of the contigs also do not have a taxonomic assignment (Table S1), and although identification of all abundant genera was consistent with previous 16S rRNA studies (21, 22, 34), some bacteria were absent or underrepresented in the metagenomes compared to 16S rRNA surveys and vice versa (Table S3). The lack of identification of the presence of members of the TG3 phylum in the metagenome is likely an artifact, as the phylum lies within the *Fibrobacteres* in the NCBI sequence database we used for taxonomic identification. The genera *Chitinispirillum* and *Ruminiclostridium* we identified in the metagenomes were absent in 16S rRNA analyses (21, 22, 34) because there were no rRNA gene references for these two genera in the database at the time they were analyzed and published (52). Also, the lack of appropriate reference genomes for termite-associated strains limits the classification of the metagenome to genus level, which is likely the cause for the underrepresentation of the genus *Alistipes*. Some *Alistipes* sequences may not have been identified because they do not match to the reference genome which are mostly isolated from the human microbiome (53–56). Thus, limited by reference-dependent methods that require closely related sequences in the public databases, the contribution of cell wall-degrading enzymes of some taxa is likely to be underestimated. Deeper metagenome sequencing that can enable binning of sequences to improve taxonomic classification and functional predictions, coupled with functional studies of the gut-compartment-specific expression of bacterial enzymes, are thus warranted.

10.1128/mSphere.00165-19.3 TABLE S3 Comparison of the taxonomic assignments of 16S rRNA studies (21, 22, 34) and metagenomic sequences (this study) at phylum (top) and genus (bottom) levels. Taxonomy classifications with more than 0.1% of the total abundance are shown. Taxonomy with no 16S rRNA gene references are marked as “NA.” Download Table S3, XLSX file, 0.02 MB. Copyright © 2019 Hu et al. 2019 Hu et al. This content is distributed under the terms of the Creative Commons Attribution 4.0 International license.

Similarities in host diet have been shown to drive convergence in the functional potential of gut microbes in other organisms (57, 58). Selection for particular physiological traits may, however, not necessarily be directly linked to specific phylogenetic groups of microbes. Exploring communities associated with diverse fungus-growing hosts (and their associated fungi) would allow us to explicitly test for convergent evolution of mycolytic microbial communities, even if these were likely comprised of different microbial consortia. A number of other insects utilize fungus material as a nutrient source, including fungus-growing ants (59, 60), some *Drosophila* species (61), the Malaysian mushroom-harvesting ant *Euprenolepis* (62), *Sirex* wood wasps (63, 64), and ambrosia beetles (65–67). Different bacteria are likely to be involved, but predictions would be that predominantly fungal diets should select for microbial communities with comparable mycolytic capacities. Recent work on mycophagous *Drosophila* supports that this may be so. Bost et al. (68) demonstrated that gut bacteria in mycophagous *Drosophila* are implicated in fungal cell wall metabolism, with cysteine and methionine metabolism enzymes originating from *Bacteroidetes*, *Firmicutes*, and *Proteobacteria* gut microbiota members, phyla that are also abundant and mycolytic in fungus-growing termites.

The prevalence of microbial communities with ample mycolytic capacities in the guts of fungus-growing termite species suggests that the adoption of a fungal diet has been associated with a functional and compositional change in gut microbial communities at the onset of fungiculture in termites. The high relative abundance of fungal cell wall-degrading enzymes indicates adaptations to the decomposition of fungal diet, which is consistent with this capacity being absent or less in termites with predominantly plant-based diets. An exception is wood-feeding termites, for which wood-degrading fungi may also comprise an appreciable component of the termite diet. To improve our understanding of the link between the digestive function and the gut microbes in fungus-growing termites, further work will be needed to elucidate whether the functional capacities of the gut microbiota reflect the amount of fungal biomass in the diet and differences in properties of the fungal species fed on. Estimates of bacterial densities and enzyme activities are also needed to fully understand the capacity of biomass degradation in termite gut. Metagenomic analysis, including more fungus-growing termite species, improved coverage, and longer reads, paired with reference-free taxonomy binning methods and functional annotation, will also largely enhance our understanding of the property of gut microbes in a functional angle.

## MATERIALS AND METHODS

### *Odontotermes* sp. collection.

Termites from an *Odontotermes* sp. colony (code Od127) were collected at the Experimental Farm of the University of Pretoria, Pretoria, South Africa (−25.742700, 28.256517). The species identity of this colony had been previously established as by mitochondrial gene COII barcoding (22). Fifty old major workers were sampled, and entire guts were dissected and pooled in a 1.5-ml Eppendorf tube and stored at –80°C until DNA extraction.

### Gut microbiota DNA extraction.

Guts were ground in liquid nitrogen, and DNA was extracted using DNeasy blood and tissue kits (Qiagen, Hilden, Germany) according to the manufacturer’s description, except that a chloroform extraction step was included after incubation with protease K. After proteinase K digestion, 1 volume chloroform-isoamyl alcohol (24/1) was added. The tubes were incubated for 15 min on a slowly rotating wheel and then centrifuged at 3,000 × *g* for 10 min. The supernatant was transferred to spin columns, and the remainder of the manufacturer’s protocol was followed. The quality and purity of samples were determined using NanoDrop (Thermo Scientific, Wilmington, DE).

### Metagenome sequencing and assembly.

DNA was sheared to ∼350-bp fragments, end repaired, A tailed, and ligated with Illumina paired-end adaptors (Illumina). The ligated fragments were selected from the desired size on agarose gels and amplified by ligation-mediated PCR, and libraries were sequenced with 150-bp read lengths on an Illumina HiSeq2500. The quality of raw sequencing reads was assessed before assembly. Reads containing the adaptor, >10% N, or >50% low-quality bases (Q-score ≥ 5) were removed. To exclude sequences from *Termitomyces* and the termite hosts, quality-controlled reads were mapped to the *M. natalensis* and *Termitomyces* genomes (24) using the Burrows-Wheeler Aligner v0.7.15 (69) BWA-MEM algorithm; any aligned reads were filtered.

Clean reads were assembled by IDBA-UD v1.1.2 (70, 71) with an iterative set up from k-mer size of 19 to 99 at step of 10 (–pre_correction –mink 19 –maxk 99 –step 10). Unassembled reads were picked out by mapping reads back to the initial assembly and assembled separately with the same setup. Redundancies of sequences from the same organism within the metagenome were removed by clustering all contigs at 95% identity with CD-hit v4.6.6 (72), and only the longest contig per cluster was kept. For comparison, high-quality reads of *M. natalensis* old major worker gut microbiota from Poulsen et al. (24) were also reassembled using the same procedure. Genes in both assemblies were predicted by Prodigal v2.6.3 (73) with metagenomics parameters (–c –p meta).

### Non-fungus-growing termite gut metagenomes.

We obtained seven published non-fungus-growing termite gut metagenomes. These included a dung feeder, *Amitermes wheeleri* (33); two wood feeders, *Nasutitermes corniger* and *Microcerotermes parvus*; a litter feeder, *Cornitermes* sp.; two humus feeders, *Termes hospes* and *Neocapritermes taracua*; and a soil feeder, *Cubitermes ugandensis* (34). Contigs and protein coding genes were downloaded from JGI IMG/M (https://img.jgi.doe.gov/) (Table 2).

**TABLE 2:** Summary of metagenome data

Termite species	Diet	Reference	Accession no.	Sample data size (Gbp)	Assembled bases (%)	Assembly size (Mbp)	Assembly *N*_50_ (bp)	No. of genes
*Odontotermes* sp.	Fungus	NA^*a*^	PRJNA476694	8.6	57.9	797.15	1,133	753,265
Macrotermes natalensis	Fungus	24	PRJNA193472	8.5	50.9	498.71	1,569	486,207
Amitermes wheeleri	Dung	33	PRJNA173365	0.3	57.7	139.30	512	321,461
Nasutitermes corniger	Wood	34	PRJNA366361	46.8	20.5	634.52	980	1,338,688
Microcerotermes parvus	Wood	34	PRJNA271983	43.2	25.0	712.35	945	1,419,719
Cornitermes sp.	Litter	34	PRJNA405701	45.9	25.3	131.61	519	2,945,692
Termes hospes	Humus	34	PRJNA405704	34.2	16.7	1,212.37	424	2,975,319
Neocapritermes taracua	Humus	34	PRJNA366256	28.3	8.8	885.58	454	2,115,406
Cubitermes ugandensis	Soil	34	PRJNA366375	32.0	10.8	1,121.61	475	3,061,751

a NA, not applicable.

### Relative abundances of contigs within metagenomes.

Clean reads of fungus-growing termites gut microbiota were mapped to the contigs by Bowtie v2.2.9 (74) with subsequent masked duplication, taking the best match for each read. Contig coverage was first estimated by the contig length and the number of mapped reads per contigs. Mapped read numbers were scaled to an equivalent of 10 Gb of sequence per metagenome, after which the relative abundance of each contig was calculated as the coverage of the contig divided by the sum of coverages of all contigs (cf. reference 75). For non-fungus-growing termites, coverage information of contigs was obtained from JGI (76, 77), and the relative abundances were estimated as described above.

### Metagenome taxonomic assignment.

Taxonomic assignment of protein-coding genes was carried out using Diamond (18) alignment against the NR database in NCBI. Alignments with E values >1e–5 and sequences with identities <30% were removed. Taxonomic information of the top hit was assigned to each gene. Contigs referring to taxonomical levels were determined by a modified lowest common ancestor (LCA)-based algorithm implemented in MEGAN (78). Taxonomic classification supported by <10% of the genes on each contig were first filtered, and the LCAs for the taxonomic classification of the rest of the genes were assigned to the contig. The relative abundance of contigs belonging to the same taxonomic group was summed up to represent the taxonomic composition of that taxonomic group in the microbiota. Taxonomic composition at the phylum level for metagenomes was centered log ratio (CLR) transformed and then compared and visualized by principle component analysis (PCA) (79) using R v3.3.2 (80).

### Carbohydrate-active enzyme analysis.

We classified genes in CAZyme families by searching against CAZyme hidden Markov profiles in the dbCAN database (v7; CAZyDB accessed 31 July 2018 [81]) using HMMer v3.1b (E ≤ 1e–5) (82). The longest matched profile was selected if CAZyme domain overlapped with peptide pattern recognition using HotPep (36–38). CAZyme family classifications that were supported by both approaches were kept. The composition of GH families in termite species was CLR transformed, and the differences between termite species were visualized using PCA (79) in R v3.3.2 (80). Enzyme functions of genes in each CAZyme family were determined by BLASTp searches (E ≤ 1e–5) against the ExPASy enzyme records, CCD searches against the COG database (83), and GhostKOALA searches using the KEGG database online tool (84). Genes coding for enzymes related to fungal and plant cell wall degradation were selected, and their substrate targets and bacterial taxonomy were assigned and manually checked. To compare the fungal and plant cell wall-targeting enzymes across termite species, the relative abundance profiles were hierarchically clustered and are shown as a dendrogram in Fig. 2C. For each enzyme, the relative abundances were compared to the average across the nine termite species, and the fold change was log_2_ transformed and is presented in a heatmap in Fig. 2C.

### Data availability.

Clean reads and metagenome assembly have been submitted to the SRA and GenBank under BioProject accession numbers PRJNA476694 and PRJNA193472.
