# Supplementary material for: Fungiculture in Termites Is Associated with a Mycolytic Gut Bacterial Community
Source: mSphere. 2019 May 15;4(3):e00165-19. doi: 10.1128/mSphere.00165-19 (PMC6520439; doi:10.1128/mSphere.00165-19)
Supplement: TABLE S1 [file mSphere.00165-19-st001.docx]

| **Level** | **Taxa** | **Mn** | **Od** | **Cu** | **Aw** | **Nt** | **Th** | **Co** | **Mp** | **Nc** |
| --- | --- | --- | --- | --- | --- | --- | --- | --- | --- | --- |
| **Phylum** | Spirochaetes | 0.034 | 0.069 | 0.010 | 0.211 | 0.109 | 0.084 | 0.336 | 0.503 | 0.462 |
|  | Firmicutes | 0.259 | 0.236 | 0.091 | 0.296 | 0.221 | 0.251 | 0.163 | 0.129 | 0.122 |
|  | Bacteroidetes | 0.300 | 0.249 | 0.126 | 0.042 | 0.146 | 0.089 | 0.038 | 0.049 | 0.041 |
|  | Proteobacteria | 0.079 | 0.076 | 0.093 | 0.068 | 0.096 | 0.114 | 0.033 | 0.040 | 0.043 |
|  | Fibrobacteres | 0.001 | 0.006 | 0.001 | 0.015 | 0.001 | 0.001 | 0.045 | 0.080 | 0.129 |
|  | Planctomycetes | 0.033 | 0.017 | 0.017 | 0.010 | 0.035 | 0.056 | 0.014 | 0.003 | 0.002 |
|  | Actinobacteria | 0.011 | 0.019 | 0.007 | 0.008 | 0.022 | 0.008 | 0.003 | 0.005 | 0.003 |
|  | Synergistetes | 0.008 | 0.006 | 0.001 | 0.016 | 0.007 | 0.018 | 0.001 | 0.001 | 0.001 |
|  | Acidobacteria | 0.001 | 0.001 | 0.001 | 0.004 | 0.012 | 0.003 | 0.001 | 0.005 | 0.008 |
|  | Other | 0.021 | 0.023 | 0.035 | 0.049 | 0.042 | 0.043 | 0.024 | 0.039 | 0.036 |
|  | Unassigned | 0.254 | 0.298 | 0.618 | 0.280 | 0.311 | 0.334 | 0.343 | 0.148 | 0.152 |
| **Class** | Spirochaetia | 0.033 | 0.067 | 0.008 | 0.206 | 0.105 | 0.080 | 0.331 | 0.495 | 0.454 |
|  | Clostridia | 0.230 | 0.206 | 0.072 | 0.245 | 0.186 | 0.212 | 0.129 | 0.095 | 0.092 |
|  | Bacteroidia | 0.261 | 0.208 | 0.069 | 0.023 | 0.099 | 0.055 | 0.019 | 0.027 | 0.019 |
|  | Deltaproteobacteria | 0.028 | 0.030 | 0.026 | 0.035 | 0.063 | 0.049 | 0.012 | 0.015 | 0.018 |
|  | Bacilli | 0.011 | 0.013 | 0.011 | 0.029 | 0.017 | 0.021 | 0.015 | 0.015 | 0.013 |
|  | Betaproteobacteria | 0.017 | 0.017 | 0.043 | 0.009 | 0.009 | 0.033 | 0.004 | 0.005 | 0.005 |
|  | Fibrobacteria | 0.000 | 0.003 | 0.000 | 0.006 | 0.000 | 0.001 | 0.006 | 0.039 | 0.073 |
|  | Gammaproteobacteria | 0.013 | 0.010 | 0.015 | 0.015 | 0.013 | 0.017 | 0.010 | 0.012 | 0.011 |
|  | Chitinispirillia | 0.000 | 0.000 | 0.000 | 0.008 | 0.000 | 0.000 | 0.033 | 0.026 | 0.029 |
|  | Planctomycetia | 0.011 | 0.008 | 0.009 | 0.006 | 0.019 | 0.027 | 0.008 | 0.002 | 0.001 |
|  | Actinobacteria | 0.006 | 0.015 | 0.004 | 0.007 | 0.013 | 0.006 | 0.002 | 0.004 | 0.002 |
|  | Synergistia | 0.008 | 0.006 | 0.001 | 0.016 | 0.007 | 0.018 | 0.001 | 0.001 | 0.001 |
|  | Planctomycetes_bacterium_RBG_16_64_12 | 0.003 | 0.002 | 0.003 | 0.002 | 0.007 | 0.010 | 0.003 | 0.000 | 0.000 |
|  | Fibrobacteres_bacterium_CG2_30_45_31 | 0.000 | 0.002 | 0.000 | 0.001 | 0.000 | 0.000 | 0.001 | 0.007 | 0.012 |
|  | Other | 0.060 | 0.078 | 0.089 | 0.110 | 0.128 | 0.106 | 0.058 | 0.084 | 0.081 |
|  | Unassigned | 0.318 | 0.336 | 0.649 | 0.283 | 0.333 | 0.364 | 0.369 | 0.174 | 0.190 |
| **Order** | Spirochaetales | 0.033 | 0.066 | 0.203 | 0.007 | 0.104 | 0.079 | 0.329 | 0.492 | 0.451 |
|  | Clostridiales | 0.223 | 0.200 | 0.237 | 0.068 | 0.179 | 0.205 | 0.124 | 0.092 | 0.090 |
|  | Bacteroidales | 0.259 | 0.205 | 0.023 | 0.067 | 0.096 | 0.054 | 0.019 | 0.026 | 0.018 |
|  | Fibrobacterales | 0.000 | 0.003 | 0.006 | 0.000 | 0.000 | 0.001 | 0.006 | 0.040 | 0.073 |
|  | Bacillales | 0.009 | 0.010 | 0.024 | 0.008 | 0.014 | 0.015 | 0.013 | 0.013 | 0.011 |
|  | Desulfovibrionales | 0.014 | 0.013 | 0.015 | 0.005 | 0.033 | 0.007 | 0.003 | 0.003 | 0.005 |
|  | Chitinispirillales | 0.000 | 0.000 | 0.008 | 0.000 | 0.000 | 0.000 | 0.033 | 0.026 | 0.029 |
|  | Planctomycetales | 0.010 | 0.007 | 0.005 | 0.009 | 0.018 | 0.026 | 0.007 | 0.001 | 0.001 |
|  | Synergistales | 0.008 | 0.006 | 0.016 | 0.001 | 0.007 | 0.018 | 0.001 | 0.001 | 0.001 |
|  | Myxococcales | 0.001 | 0.001 | 0.001 | 0.011 | 0.001 | 0.028 | 0.003 | 0.001 | 0.001 |
|  | Rhodocyclales | 0.001 | 0.003 | 0.001 | 0.012 | 0.003 | 0.020 | 0.001 | 0.001 | 0.000 |
|  | Candidatus_Adiutrix_intracellularis | 0.003 | 0.006 | 0.007 | 0.000 | 0.012 | 0.002 | 0.002 | 0.003 | 0.003 |
|  | Planctomycetes_bacterium_RBG_16_64_12 | 0.003 | 0.002 | 0.002 | 0.003 | 0.007 | 0.011 | 0.003 | 0.000 | 0.000 |
|  | Fibrobacteres_bacterium_CG2_30_45_31 | 0.000 | 0.002 | 0.001 | 0.000 | 0.000 | 0.000 | 0.001 | 0.006 | 0.012 |
|  | Other | 0.099 | 0.121 | 0.167 | 0.134 | 0.180 | 0.159 | 0.083 | 0.117 | 0.111 |
|  | Unassigned | 0.336 | 0.356 | 0.284 | 0.673 | 0.344 | 0.375 | 0.373 | 0.179 | 0.194 |
| **Family** | Spirochaetaceae | 0.032 | 0.066 | 0.007 | 0.204 | 0.104 | 0.079 | 0.329 | 0.492 | 0.452 |
|  | Ruminococcaceae | 0.036 | 0.049 | 0.013 | 0.074 | 0.040 | 0.056 | 0.019 | 0.017 | 0.017 |
|  | Clostridiaceae | 0.026 | 0.029 | 0.016 | 0.050 | 0.034 | 0.030 | 0.020 | 0.020 | 0.019 |
|  | Lachnospiraceae | 0.025 | 0.021 | 0.013 | 0.045 | 0.022 | 0.027 | 0.019 | 0.018 | 0.018 |
|  | Porphyromonadaceae | 0.040 | 0.049 | 0.022 | 0.005 | 0.044 | 0.019 | 0.005 | 0.008 | 0.004 |
|  | Rikenellaceae | 0.107 | 0.065 | 0.002 | 0.001 | 0.003 | 0.002 | 0.001 | 0.001 | 0.001 |
|  | Fibrobacteraceae | 0.000 | 0.003 | 0.000 | 0.006 | 0.000 | 0.001 | 0.006 | 0.039 | 0.073 |
|  | Bacteroidaceae | 0.043 | 0.024 | 0.009 | 0.005 | 0.013 | 0.009 | 0.004 | 0.004 | 0.003 |
|  | Chitinispirillaceae | 0.000 | 0.000 | 0.000 | 0.008 | 0.000 | 0.000 | 0.033 | 0.026 | 0.029 |
|  | Desulfovibrionaceae | 0.013 | 0.012 | 0.004 | 0.014 | 0.032 | 0.006 | 0.002 | 0.003 | 0.005 |
|  | Peptococcaceae | 0.008 | 0.011 | 0.008 | 0.016 | 0.017 | 0.013 | 0.006 | 0.007 | 0.006 |
|  | Planctomycetaceae | 0.007 | 0.005 | 0.006 | 0.004 | 0.014 | 0.022 | 0.006 | 0.001 | 0.000 |
|  | Paenibacillaceae | 0.004 | 0.005 | 0.004 | 0.012 | 0.006 | 0.007 | 0.006 | 0.007 | 0.005 |
|  | Synergistaceae | 0.008 | 0.006 | 0.001 | 0.015 | 0.007 | 0.017 | 0.001 | 0.001 | 0.001 |
|  | Eubacteriaceae | 0.003 | 0.003 | 0.003 | 0.015 | 0.008 | 0.006 | 0.004 | 0.004 | 0.003 |
|  | Rhodocyclaceae | 0.001 | 0.003 | 0.012 | 0.001 | 0.003 | 0.021 | 0.001 | 0.001 | 0.000 |
|  | Candidatus_Adiutrix_intracellularis | 0.003 | 0.006 | 0.000 | 0.007 | 0.012 | 0.002 | 0.002 | 0.004 | 0.003 |
|  | Planctomycetes_bacterium_RBG_16_64_12 | 0.003 | 0.002 | 0.003 | 0.002 | 0.007 | 0.010 | 0.003 | 0.000 | 0.000 |
|  | Fibrobacteres_bacterium_CG2_30_45_31 | 0.000 | 0.002 | 0.000 | 0.001 | 0.000 | 0.000 | 0.001 | 0.007 | 0.012 |
|  | Archangiaceae | 0.000 | 0.000 | 0.006 | 0.000 | 0.000 | 0.014 | 0.001 | 0.000 | 0.000 |
|  | Other | 0.143 | 0.164 | 0.171 | 0.225 | 0.244 | 0.211 | 0.108 | 0.145 | 0.135 |
|  | Unassigned | 0.496 | 0.476 | 0.699 | 0.290 | 0.388 | 0.448 | 0.423 | 0.196 | 0.214 |
| **Genus** | Other | 0.216 | 0.249 | 0.232 | 0.358 | 0.345 | 0.321 | 0.159 | 0.195 | 0.181 |
|  | Treponema | 0.031 | 0.064 | 0.007 | 0.197 | 0.102 | 0.077 | 0.322 | 0.482 | 0.444 |
|  | Alistipes | 0.083 | 0.049 | 0.002 | 0.001 | 0.002 | 0.002 | 0.001 | 0.001 | 0.000 |
|  | Bacteroides | 0.042 | 0.024 | 0.009 | 0.005 | 0.013 | 0.009 | 0.003 | 0.004 | 0.003 |
|  | Clostridium | 0.019 | 0.022 | 0.012 | 0.039 | 0.026 | 0.022 | 0.016 | 0.016 | 0.015 |
|  | Dysgonomonas | 0.018 | 0.010 | 0.003 | 0.001 | 0.005 | 0.003 | 0.001 | 0.001 | 0.001 |
|  | Desulfovibrio | 0.010 | 0.009 | 0.004 | 0.012 | 0.028 | 0.005 | 0.002 | 0.003 | 0.004 |
|  | Parabacteroides | 0.006 | 0.009 | 0.005 | 0.001 | 0.013 | 0.006 | 0.001 | 0.002 | 0.000 |
|  | Candidatus Adiutrix | 0.003 | 0.006 | 0.000 | 0.008 | 0.012 | 0.002 | 0.002 | 0.004 | 0.003 |
|  | Ruminococcus | 0.007 | 0.004 | 0.004 | 0.027 | 0.008 | 0.009 | 0.003 | 0.005 | 0.005 |
|  | Ruminiclostridium | 0.003 | 0.004 | 0.003 | 0.019 | 0.006 | 0.007 | 0.004 | 0.006 | 0.005 |
|  | Fibrobacter | 0.000 | 0.003 | 0.000 | 0.006 | 0.000 | 0.001 | 0.006 | 0.040 | 0.073 |
|  | Eubacterium | 0.002 | 0.002 | 0.002 | 0.013 | 0.006 | 0.005 | 0.003 | 0.003 | 0.003 |
|  | Planctomycetes_bacterium_RBG_16_64_12 | 0.003 | 0.002 | 0.003 | 0.002 | 0.007 | 0.010 | 0.003 | 0.000 | 0.000 |
|  | Fibrobacteres_bacterium_CG2_30_45_31 | 0.000 | 0.002 | 0.000 | 0.001 | 0.000 | 0.000 | 0.001 | 0.007 | 0.012 |
|  | Sporobacter | 0.000 | 0.001 | 0.001 | 0.011 | 0.014 | 0.012 | 0.005 | 0.001 | 0.001 |
|  | Chitinispirillum | 0.000 | 0.000 | 0.000 | 0.008 | 0.000 | 0.000 | 0.033 | 0.026 | 0.029 |
|  | Azovibrio | 0.000 | 0.000 | 0.001 | 0.000 | 0.000 | 0.014 | 0.000 | 0.000 | 0.000 |
|  | Unassigned | 0.555 | 0.540 | 0.712 | 0.293 | 0.411 | 0.496 | 0.434 | 0.205 | 0.221 |
