# Supplementary material for: Fungiculture in Termites Is Associated with a Mycolytic Gut Bacterial Community
Source: mSphere. 2019 May 15;4(3):e00165-19. doi: 10.1128/mSphere.00165-19 (PMC6520439; doi:10.1128/mSphere.00165-19)
Supplement: TABLE S2 [file mSphere.00165-19-st002.docx]

| **Glycoside Hydrolase family** | **Mn** | **Od** | **Mp** | **Nc** | **Cu** | **Th** | **Nt** | **Co** | **Aw** | **Fold change between fungus vs. non-fungus growing termites** | **Enzyme List** |
| --- | --- | --- | --- | --- | --- | --- | --- | --- | --- | --- | --- |
| GH59 | 7.09E-06 | 1.30E-05 | 0.00E+00 | 0.00E+00 | 1.70E-07 | 3.99E-07 | 1.37E-06 | 0.00E+00 | 0.00E+00 | 36.23 | β-galactosidase (EC 3.2.1.23); galactocerebrosidase (EC 3.2.1.46) |
| GH125 | 3.98E-05 | 3.45E-05 | 1.14E-06 | 1.95E-06 | 1.13E-06 | 2.10E-06 | 1.07E-06 | 1.40E-07 | 0.00E+00 | 34.57 | exo-α-1,6-mannosidase (EC 3.2.1.-) |
| GH108 | 7.23E-05 | 8.95E-05 | 1.99E-06 | 2.65E-06 | 7.68E-07 | 2.49E-06 | 3.79E-06 | 1.23E-06 | 9.21E-06 | 25.58 | lysozyme (EC 3.2.1.17) |
| GH97 | 1.45E-04 | 1.03E-04 | 4.56E-06 | 2.64E-06 | 4.77E-06 | 6.68E-06 | 1.58E-05 | 2.52E-06 | 0.00E+00 | 23.41 | glucoamylase (EC 3.2.1.3); α-glucosidase (EC 3.2.1.20); α-galactosidase (EC 3.2.1.22) |
| GH89 | 4.22E-05 | 1.52E-05 | 4.14E-07 | 3.75E-06 | 1.33E-06 | 2.23E-07 | 4.37E-06 | 9.42E-08 | 0.00E+00 | 19.73 | α-N-acetylglucosaminidase (EC 3.2.1.50) |
| GH114 | 6.17E-06 | 1.19E-05 | 5.49E-07 | 1.72E-07 | 0.00E+00 | 1.15E-07 | 2.08E-06 | 6.79E-07 | 0.00E+00 | 17.59 | endo-α-1,4-polygalactosaminidase (EC 3.2.1.109) |
| GH92 | 2.88E-04 | 1.52E-04 | 1.13E-05 | 7.80E-06 | 1.01E-05 | 1.77E-05 | 5.54E-05 | 3.25E-06 | 0.00E+00 | 14.58 | mannosyl-oligosaccharide α-1,2-mannosidase (EC 3.2.1.113); mannosyl-oligosaccharide α-1,3-mannosidase (EC 3.2.1.-); mannosyl-oligosaccharide α-1,6-mannosidase (EC 3.2.1.-); α-mannosidase (EC 3.2.1.24); α-1,2-mannosidase (EC 3.2.1.-); α-1,3-mannosidase (EC 3.2.1.-); α-1,4-mannosidase (EC 3.2.1.-); mannosyl-1-phosphodiester α-1,P-mannosidase (EC 3.2.1.-) |
| GH25 | 2.23E-04 | 8.70E-05 | 1.70E-05 | 1.48E-05 | 9.91E-06 | 8.83E-06 | 2.33E-05 | 9.36E-06 | 6.14E-06 | 12.14 | lysozyme (EC 3.2.1.17) |
| GH87 | 1.56E-05 | 6.66E-06 | 2.97E-06 | 9.58E-07 | 0.00E+00 | 7.36E-07 | 2.11E-06 | 1.25E-06 | 0.00E+00 | 9.72 | mycodextranase (EC 3.2.1.61); α-1,3-glucanase (EC 3.2.1.59) |
| GH24 | 6.17E-05 | 6.08E-05 | 2.39E-06 | 4.94E-06 | 8.34E-06 | 1.12E-05 | 5.12E-06 | 3.19E-06 | 1.54E-05 | 8.49 | lysozyme (EC 3.2.1.17) |
| GH55 | 5.90E-05 | 2.66E-05 | 2.11E-05 | 0.00E+00 | 7.01E-07 | 4.09E-06 | 8.95E-06 | 3.09E-06 | 0.00E+00 | 7.89 | exo-β-1,3-glucanase (EC 3.2.1.58); endo-β-1,3-glucanase (EC 3.2.1.39) |
| GH133 | 1.16E-04 | 1.05E-04 | 5.24E-06 | 3.79E-06 | 1.21E-05 | 1.59E-05 | 2.51E-05 | 3.69E-06 | 5.37E-05 | 6.49 | amylo-α-1,6-glucosidase (EC 3.2.1.33); |
| GH95 | 1.60E-04 | 1.35E-04 | 4.65E-05 | 4.21E-05 | 6.80E-06 | 2.44E-05 | 3.20E-05 | 1.37E-05 | 0.00E+00 | 6.22 | α-L-fucosidase (EC 3.2.1.51); α-1,2-L-fucosidase (EC 3.2.1.63); α-L-galactosidase (EC 3.2.1.-) |
| GH93 | 1.44E-05 | 1.50E-05 | 3.54E-06 | 3.89E-06 | 4.50E-07 | 2.55E-06 | 4.10E-06 | 2.59E-06 | 0.00E+00 | 6.01 | exo-α-L-1,5-arabinanase (EC 3.2.1.-) |
| GH110 | 1.54E-05 | 9.71E-06 | 6.69E-07 | 4.68E-07 | 0.00E+00 | 3.22E-06 | 9.24E-06 | 1.08E-06 | 0.00E+00 | 5.98 | α-galactosidase (EC 3.2.1.22); α-1,3-galactosidase (EC 3.2.1.-) |
| GH32 | 5.32E-05 | 6.39E-05 | 5.21E-06 | 2.90E-06 | 5.14E-06 | 9.92E-06 | 1.56E-05 | 5.09E-06 | 2.64E-05 | 5.83 | invertase (EC 3.2.1.26); endo-inulinase (EC 3.2.1.7); β-2,6-fructan 6-levanbiohydrolase (EC 3.2.1.64); endo-levanase (EC 3.2.1.65); exo-inulinase (EC 3.2.1.80); fructan β-(2,1)-fructosidase/1-exohydrolase (EC 3.2.1.153); fructan β-(2,6)-fructosidase/6-exohydrolase (EC 3.2.1.154); sucrose:sucrose 1-fructosyltransferase (EC 2.4.1.99); fructan:fructan 1-fructosyltransferase (EC 2.4.1.100); sucrose:fructan 6-fructosyltransferase (EC 2.4.1.10); fructan:fructan 6G-fructosyltransferase (EC 2.4.1.243); levan fructosyltransferase (EC 2.4.1.-); [retaining] sucrose:sucrose 6-fructosyltransferase (6-SST) (EC 2.4.1.-); cycloinulo-oligosaccharide fructanotransferase (EC 2.4.1.-) |
| GH128 | 7.74E-06 | 4.01E-05 | 8.46E-06 | 1.01E-05 | 5.63E-07 | 3.09E-06 | 1.02E-06 | 6.18E-06 | 0.00E+00 | 5.70 | β-1,3-glucanase (EC 3.2.1.39) |
| GH63 | 3.42E-05 | 8.44E-06 | 1.56E-05 | 3.61E-06 | 0.00E+00 | 1.32E-06 | 3.42E-06 | 2.51E-06 | 0.00E+00 | 5.64 | processing α-glucosidase (EC 3.2.1.106); α-1,3-glucosidase (EC 3.2.1.84); α-glucosidase (EC 3.2.1.20); mannosylglycerate α-mannosidase / mannosylglycerate hydrolase (EC 3.2.1.170) |
| GH104 | 7.94E-07 | 2.13E-06 | 1.87E-07 | 0.00E+00 | 1.08E-06 | 3.77E-07 | 1.44E-07 | 6.00E-08 | 0.00E+00 | 5.54 | peptidoglycan lytic transglycosylase (EC 3.2.1.-) |
| GH88 | 1.25E-04 | 7.38E-05 | 2.45E-05 | 1.34E-05 | 1.67E-06 | 1.45E-05 | 1.35E-05 | 1.17E-05 | 4.73E-05 | 5.51 | d-4,5-unsaturated β-glucuronyl hydrolase (EC 3.2.1.-) |
| GH35 | 3.90E-05 | 2.84E-05 | 1.10E-05 | 1.38E-05 | 1.60E-06 | 7.81E-06 | 5.55E-06 | 7.23E-06 | 0.00E+00 | 5.02 | β-galactosidase (EC 3.2.1.23); exo-β-glucosaminidase (EC 3.2.1.165); exo-β-1,4-galactanase (EC 3.2.1.-); β-1,3-galactosidase (EC 3.2.1.-) |
| GH2 | 6.24E-04 | 4.34E-04 | 2.23E-04 | 1.77E-04 | 1.82E-05 | 1.06E-04 | 9.71E-05 | 1.04E-04 | 2.12E-05 | 4.96 | β-galactosidase (EC 3.2.1.23) ; β-mannosidase (EC 3.2.1.25); β-glucuronidase (EC 3.2.1.31); α-L-arabinofuranosidase (EC 3.2.1.55); mannosylglycoprotein endo-β-mannosidase (EC 3.2.1.152); exo-β-glucosaminidase (EC 3.2.1.165) |
| GH78 | 1.95E-04 | 1.84E-04 | 8.08E-05 | 2.42E-05 | 2.26E-05 | 4.37E-05 | 8.90E-05 | 1.32E-05 | 2.46E-05 | 4.45 | α-L-rhamnosidase (EC 3.2.1.40); rhamnogalacturonan α-L-rhamnohydrolase (EC 3.2.1.174) |
| GH99 | 9.14E-05 | 9.21E-05 | 3.46E-05 | 3.43E-05 | 8.77E-06 | 2.50E-05 | 2.96E-05 | 1.52E-05 | 0.00E+00 | 4.36 | glycoprotein endo-α-1,2-mannosidase (EC 3.2.1.130); mannan endo-1,2-α-mannanase (3.2.1.-) |
| GH123 | 2.78E-05 | 1.75E-05 | 7.69E-06 | 1.61E-06 | 1.60E-06 | 1.54E-05 | 9.78E-06 | 3.05E-06 | 0.00E+00 | 4.06 | β-N-acetylgalactosaminidase (EC 3.2.1.53); glycosphingolipid β-N-acetylgalactosaminidase (EC 3.2.1.-) |
| GH105 | 1.83E-04 | 1.63E-04 | 8.54E-05 | 5.54E-05 | 2.54E-06 | 4.46E-05 | 2.92E-05 | 3.68E-05 | 4.88E-05 | 4.00 | unsaturated rhamnogalacturonyl hydrolase (EC 3.2.1.172); d-4,5-unsaturated β-glucuronyl hydrolase (EC 3.2.1.-) |
| GH17 | 4.29E-06 | 4.18E-06 | 0.00E+00 | 1.53E-07 | 1.83E-06 | 1.56E-06 | 3.68E-06 | 3.56E-07 | 0.00E+00 | 3.91 | glucan endo-1,3-β-glucosidase (EC 3.2.1.39); glucan 1,3-β-glucosidase (EC 3.2.1.58); licheninase (EC 3.2.1.73); ABA-specific β-glucosidase (EC 3.2.1.175); β-1,3-glucanosyltransglycosylase (EC 2.4.1.-) |
| GH112 | 3.23E-05 | 2.13E-05 | 1.28E-05 | 1.14E-05 | 0.00E+00 | 1.24E-05 | 9.93E-07 | 1.09E-05 | 0.00E+00 | 3.87 | lacto-N-biose phosphorylase or galacto-N-biose phosphorylase (EC 2.4.1.211); D-galactosyl-β-1,4-L-rhamnose phosphorylase (EC 2.4.1.247); galacto-N-biose/lacto-N-biose phosphorylase (EC 2.4.1.-) |
| GH28 | 1.51E-04 | 1.09E-04 | 4.00E-05 | 2.65E-05 | 7.05E-06 | 2.43E-05 | 4.90E-05 | 1.96E-05 | 7.34E-05 | 3.78 | polygalacturonase (EC 3.2.1.15); α-L-rhamnosidase (EC 3.2.1.40); exo-polygalacturonase (EC 3.2.1.67); exo-polygalacturonosidase (EC 3.2.1.82); rhamnogalacturonase (EC 3.2.1.171); rhamnogalacturonan α-1,2-galacturonohydrolase (EC 3.2.1.173); endo-xylogalacturonan hydrolase (EC 3.2.1.-) |
| GH65 | 1.36E-04 | 1.27E-04 | 5.12E-05 | 1.63E-05 | 3.82E-06 | 4.45E-05 | 1.19E-05 | 4.85E-05 | 6.75E-05 | 3.78 | α,α-trehalase (EC 3.2.1.28); maltose phosphorylase (EC 2.4.1.8); trehalose phosphorylase (EC 2.4.1.64); kojibiose phosphorylase (EC 2.4.1.230); trehalose-6-phosphate phosphorylase (EC 2.4.1.216); nigerose phosphorylase (EC 2.4.1.279); 3-O-α-glucopyranosyl-L-rhamnose phosphorylase (EC 2.4.1.282); 2-O-α-glucopyranosylglycerol: phosphate β-glucosyltransferase (EC 2.4.1.-); α-glucosyl-1,2-β-galactosyl-L-hydroxylysine α-glucosidase (EC 3.2.1.107) |
| GH51 | 1.46E-04 | 1.74E-04 | 9.83E-05 | 7.43E-05 | 7.56E-06 | 4.17E-05 | 2.46E-05 | 4.30E-05 | 9.52E-06 | 3.74 | endoglucanase (EC 3.2.1.4); endo-β-1,4-xylanase (EC 3.2.1.8); β-xylosidase (EC 3.2.1.37); α-L-arabinofuranosidase (EC 3.2.1.55) |
| GH127 | 1.05E-04 | 1.27E-04 | 3.48E-05 | 2.30E-05 | 1.21E-05 | 3.77E-05 | 4.55E-05 | 2.64E-05 | 3.71E-05 | 3.74 | β-L-arabinofuranosidase (EC 3.2.1.185); 3-C-carboxy-5-deoxy-L-xylose (aceric acid) hydrolase |
| GH29 | 2.12E-04 | 1.52E-04 | 5.28E-05 | 5.34E-05 | 3.28E-05 | 6.23E-05 | 8.35E-05 | 2.47E-05 | 6.60E-05 | 3.39 | α-L-fucosidase (EC 3.2.1.51); α-1,3/1,4-L-fucosidase (EC 3.2.1.111) |
| GH27 | 3.26E-05 | 1.60E-05 | 1.76E-05 | 1.71E-05 | 1.53E-06 | 1.22E-06 | 2.71E-06 | 4.16E-06 | 6.75E-06 | 3.33 | α-galactosidase (EC 3.2.1.22); α-N-acetylgalactosaminidase (EC 3.2.1.49); isomalto-dextranase (EC 3.2.1.94); β-L-arabinopyranosidase (EC 3.2.1.88); galactan:galactan galactosyltransferase (EC 2.4.1.-) |
| GH19 | 1.98E-05 | 1.58E-05 | 5.54E-06 | 9.38E-06 | 5.40E-06 | 5.31E-06 | 2.42E-06 | 9.46E-06 | 0.00E+00 | 3.32 | chitinase (EC 3.2.1.14); lysozyme (EC 3.2.1.17) |
| GH31 | 2.00E-04 | 1.96E-04 | 1.08E-04 | 7.27E-05 | 1.82E-05 | 5.43E-05 | 3.17E-05 | 3.72E-05 | 1.48E-04 | 2.95 | α-glucosidase (EC 3.2.1.20); α-galactosidase (EC 3.2.1.22); α-mannosidase (EC 3.2.1.24); α-1,3-glucosidase (EC 3.2.1.84); sucrase-isomaltase (EC 3.2.1.48) (EC 3.2.1.10); α-xylosidase (EC 3.2.1.177); α-glucan lyase (EC 4.2.2.13); isomaltosyltransferase (EC 2.4.1.-); oligosaccharide α-1,4-glucosyltransferase (EC 2.4.1.161); sulfoquinovosidase (EC 3.2.1.-) |
| GH76 | 4.69E-05 | 2.35E-05 | 8.15E-06 | 3.42E-05 | 4.97E-06 | 2.03E-05 | 1.07E-05 | 1.75E-06 | 6.14E-06 | 2.86 | α-1,6-mannanase (EC 3.2.1.101) |
| GH16 | 1.42E-04 | 1.23E-04 | 1.20E-04 | 4.78E-05 | 2.18E-05 | 2.20E-05 | 5.52E-05 | 1.79E-05 | 4.30E-05 | 2.84 | xyloglucan:xyloglucosyltransferase (EC 2.4.1.207); keratan-sulfate endo-1,4-β-galactosidase (EC 3.2.1.103); endo-1,3-β-glucanase (EC 3.2.1.39); endo-1,3(4)-β-glucanase (EC 3.2.1.6); licheninase (EC 3.2.1.73); β-agarase (EC 3.2.1.81); κ-carrageenase (EC 3.2.1.83); xyloglucanase (EC 3.2.1.151); endo-β-1,3-galactanase (EC 3.2.1.181); β-porphyranase (EC 3.2.1.178); hyaluronidase (EC 3.2.1.35); endo-β-1,4-galactosidase (EC 3.2.1.-); chitin β-1,6-glucanosyltransferase (EC 2.4.1.-); endo-β-1,4-galactosidase (EC 3.2.1.-) |
| GH18 | 2.99E-04 | 1.00E-04 | 1.44E-04 | 1.66E-04 | 1.02E-05 | 2.78E-05 | 3.09E-05 | 6.04E-05 | 6.11E-05 | 2.79 | chitinase (EC 3.2.1.14); lysozyme (EC 3.2.1.17); endo-β-N-acetylglucosaminidase (EC 3.2.1.96); peptidoglycan hydrolase with endo-β-N-acetylglucosaminidase specificity (EC 3.2.1.-); Nod factor hydrolase (EC 3.2.1.-); xylanase inhibitor; concanavalin B; narbonin |
| GH82 | 5.08E-06 | 3.43E-07 | 2.31E-06 | 0.00E+00 | 5.17E-07 | 2.28E-06 | 2.16E-06 | 9.89E-08 | 0.00E+00 | 2.58 | Ι-carrageenase (EC 3.2.1.157) |
| GH106 | 4.64E-05 | 5.71E-05 | 4.04E-05 | 2.17E-05 | 5.60E-06 | 1.55E-05 | 4.12E-05 | 1.81E-05 | 0.00E+00 | 2.54 | α-L-rhamnosidase (EC 3.2.1.40) |
| GH50 | 2.06E-05 | 6.87E-06 | 7.31E-06 | 1.73E-06 | 3.78E-06 | 1.27E-05 | 1.36E-05 | 3.19E-07 | 0.00E+00 | 2.44 | β-agarase (EC 3.2.1.81) |
| GH102 | 2.00E-06 | 1.57E-05 | 6.45E-07 | 8.28E-07 | 6.64E-06 | 5.24E-06 | 7.21E-06 | 1.39E-07 | 6.14E-06 | 2.31 | peptidoglycan lytic transglycosylase (EC 3.2.1.-) |
| GH20 | 2.51E-04 | 1.04E-04 | 1.50E-04 | 1.52E-04 | 1.02E-05 | 1.60E-05 | 5.22E-05 | 6.07E-05 | 9.85E-05 | 2.30 | β-hexosaminidase (EC 3.2.1.52); lacto-N-biosidase (EC 3.2.1.140); β-1,6-N-acetylglucosaminidase) (EC 3.2.1.-); β-6-SO3-N-acetylglucosaminidase (EC 3.2.1.-) |
| GH117 | 1.58E-05 | 3.82E-06 | 9.70E-06 | 1.90E-06 | 2.11E-06 | 6.89E-06 | 7.28E-06 | 2.36E-06 | 0.00E+00 | 2.27 | α-1,3-L-neoagarooligosaccharide hydrolase (EC 3.2.1.-); α-1,3-L-neoagarobiase / neoagarobiose hydrolase (EC 3.2.1.-) |
| GH33 | 5.69E-05 | 5.64E-05 | 5.08E-05 | 2.00E-05 | 1.47E-05 | 1.65E-05 | 3.75E-05 | 1.66E-05 | 1.93E-05 | 2.26 | sialidase or neuraminidase (EC 3.2.1.18); trans-sialidase (EC 2.4.1.-); 2-keto-3-deoxynononic acid hydrolase (EC 3.2.1.-); anhydrosialidase (EC 4.2.2.15); 3-deoxy-D-manno-octulosonic-acid hydrolase (EC 3.2.1.-) |
| GH67 | 4.24E-05 | 6.59E-05 | 6.70E-05 | 4.43E-05 | 1.96E-06 | 9.16E-06 | 5.68E-06 | 3.99E-05 | 0.00E+00 | 2.25 | α-glucuronidase (EC 3.2.1.139); xylan α-1,2-glucuronidase (EC 3.2.1.131) |
| GH36 | 4.98E-05 | 5.80E-05 | 2.07E-05 | 4.08E-05 | 7.59E-06 | 2.16E-05 | 3.17E-05 | 2.65E-05 | 2.76E-05 | 2.14 | α-galactosidase (EC 3.2.1.22); α-N-acetylgalactosaminidase (EC 3.2.1.49); stachyose synthase (EC 2.4.1.67); raffinose synthase (EC 2.4.1.82) |
| GH73 | 2.20E-04 | 1.47E-04 | 1.92E-04 | 1.52E-04 | 2.27E-05 | 4.25E-05 | 6.24E-05 | 7.67E-05 | 9.79E-05 | 1.99 | lysozyme (EC 3.2.1.17); mannosyl-glycoprotein endo-β-N-acetylglucosaminidase (EC 3.2.1.96); peptidoglycan hydrolase with endo-β-N-acetylglucosaminidase specificity (EC 3.2.1.-) |
| GH109 | 9.59E-04 | 8.62E-04 | 4.66E-04 | 3.01E-04 | 2.55E-04 | 6.84E-04 | 8.36E-04 | 2.92E-04 | 4.08E-04 | 1.97 | α-N-acetylgalactosaminidase (EC 3.2.1.49) |
| GH43 | 6.34E-04 | 5.57E-04 | 5.48E-04 | 4.73E-04 | 3.63E-05 | 1.59E-04 | 1.09E-04 | 2.74E-04 | 5.49E-04 | 1.94 | β-xylosidase (EC 3.2.1.37); α-L-arabinofuranosidase (EC 3.2.1.55); arabinanase (EC 3.2.1.99); xylanase (EC 3.2.1.8); galactan 1,3-β-galactosidase (EC 3.2.1.145); α-1,2-L-arabinofuranosidase (EC 3.2.1.-); exo-α-1,5-L-arabinofuranosidase (EC 3.2.1.-); [inverting] exo-α-1,5-L-arabinanase (EC 3.2.1.-); β-1,3-xylosidase (EC 3.2.1.-) |
| GH23 | 6.00E-04 | 5.81E-04 | 4.89E-04 | 5.61E-04 | 8.52E-05 | 1.72E-04 | 2.03E-04 | 2.38E-04 | 4.01E-04 | 1.92 | lysozyme type G (EC 3.2.1.17); peptidoglycan lyase (EC 4.2.2.n1) also known in the literature as peptidoglycan lytic transglycosylase; chitinase (EC 3.2.1.14) |
| GH103 | 9.03E-06 | 2.23E-05 | 3.56E-06 | 4.81E-06 | 7.96E-06 | 1.01E-05 | 1.60E-05 | 1.47E-06 | 1.75E-05 | 1.79 | peptidoglycan lytic transglycosylase (EC 3.2.1.-) |
| GH15 | 2.44E-06 | 4.32E-06 | 1.30E-06 | 4.26E-07 | 1.79E-06 | 2.28E-06 | 8.27E-06 | 1.50E-07 | 0.00E+00 | 1.66 | glucoamylase (EC 3.2.1.3); glucodextranase (EC 3.2.1.70); α,α-trehalase (EC 3.2.1.28); dextran dextrinase (EC 2.4.1.2) |
| GH1 | 9.10E-05 | 1.09E-04 | 1.17E-04 | 9.66E-05 | 3.68E-06 | 3.58E-05 | 2.73E-05 | 6.79E-05 | 8.04E-05 | 1.64 | β-glucosidase (EC 3.2.1.21); β-galactosidase (EC 3.2.1.23); β-mannosidase (EC 3.2.1.25); β-glucuronidase (EC 3.2.1.31); β-xylosidase (EC 3.2.1.37); β-D-fucosidase (EC 3.2.1.38); phlorizin hydrolase (EC 3.2.1.62); exo-β-1,4-glucanase (EC 3.2.1.74); 6-phospho-β-galactosidase (EC 3.2.1.85); 6-phospho-β-glucosidase (EC 3.2.1.86); strictosidine β-glucosidase (EC 3.2.1.105); lactase (EC 3.2.1.108); amygdalin β-glucosidase (EC 3.2.1.117); prunasin β-glucosidase (EC 3.2.1.118); vicianin hydrolase (EC 3.2.1.119); raucaffricine β-glucosidase (EC 3.2.1.125); thioglucosidase (EC 3.2.1.147); β-primeverosidase (EC 3.2.1.149); isoflavonoid 7-O-β-apiosyl-β-glucosidase (EC 3.2.1.161); ABA-specific β-glucosidase (EC 3.2.1.175); DIMBOA β-glucosidase (EC 3.2.1.182); β-glycosidase (EC 3.2.1.-); hydroxyisourate hydrolase (EC 3.-.-.-) |
| GH9 | 1.90E-04 | 1.30E-04 | 2.92E-04 | 2.58E-04 | 1.21E-05 | 2.37E-05 | 2.85E-05 | 9.45E-05 | 3.71E-05 | 1.50 | endoglucanase (EC 3.2.1.4); endo-β-1,3(4)-glucanase / lichenase-laminarinase (EC 3.2.1.6); β-glucosidase (EC 3.2.1.21); lichenase / endo-β-1,3-1,4-glucanase (EC 3.2.1.73); exo-β-1,4-glucanase / cellodextrinase (EC 3.2.1.74); cellobiohydrolase (EC 3.2.1.91); xyloglucan-specific endo-β-1,4-glucanase / endo-xyloglucanase (EC 3.2.1.151); exo-β-glucosaminidase (EC 3.2.1.165) |
| GH13 | 6.08E-04 | 5.93E-04 | 6.69E-04 | 8.12E-04 | 5.47E-05 | 2.83E-04 | 2.41E-04 | 3.35E-04 | 4.00E-04 | 1.50 | α-amylase (EC 3.2.1.1); pullulanase (EC 3.2.1.41); cyclomaltodextrin glucanotransferase (EC 2.4.1.19); cyclomaltodextrinase (EC 3.2.1.54); trehalose-6-phosphate hydrolase (EC 3.2.1.93); oligo-α-glucosidase (EC 3.2.1.10); maltogenic amylase (EC 3.2.1.133); neopullulanase (EC 3.2.1.135); α-glucosidase (EC 3.2.1.20); maltotetraose-forming α-amylase (EC 3.2.1.60); isoamylase (EC 3.2.1.68); glucodextranase (EC 3.2.1.70); maltohexaose-forming α-amylase (EC 3.2.1.98); maltotriose-forming α-amylase (EC 3.2.1.116); branching enzyme (EC 2.4.1.18); trehalose synthase (EC 5.4.99.16); 4-α-glucanotransferase (EC 2.4.1.25); maltopentaose-forming α-amylase (EC 3.2.1.-) ; amylosucrase (EC 2.4.1.4) ; sucrose phosphorylase (EC 2.4.1.7); malto-oligosyltrehalose trehalohydrolase (EC 3.2.1.141); isomaltulose synthase (EC 5.4.99.11); malto-oligosyltrehalose synthase (EC 5.4.99.15); amylo-α-1,6-glucosidase (EC 3.2.1.33); α-1,4-glucan: phosphate α-maltosyltransferase (EC 2.4.99.16); 6â€²-P-sucrose phosphorylase (EC 2.4.1.-); amino acid transporter |
| GH3 | 5.62E-04 | 3.87E-04 | 6.55E-04 | 6.06E-04 | 3.58E-05 | 1.68E-04 | 1.96E-04 | 2.19E-04 | 5.11E-04 | 1.39 | β-glucosidase (EC 3.2.1.21); xylan 1,4-β-xylosidase (EC 3.2.1.37); β-glucosylceramidase (EC 3.2.1.45); β-N-acetylhexosaminidase (EC 3.2.1.52); α-L-arabinofuranosidase (EC 3.2.1.55); glucan 1,3-β-glucosidase (EC 3.2.1.58); glucan 1,4-β-glucosidase (EC 3.2.1.74); isoprimeverose-producing oligoxyloglucan hydrolase (EC 3.2.1.120); coniferin β-glucosidase (EC 3.2.1.126); exo-1,3-1,4-glucanase (EC 3.2.1.-); β-N-acetylglucosaminide phosphorylases (EC 2.4.1.-) |
| GH115 | 4.53E-05 | 4.44E-05 | 9.62E-05 | 6.48E-05 | 3.03E-07 | 6.42E-06 | 3.32E-06 | 5.78E-05 | 0.00E+00 | 1.37 | xylan α-1,2-glucuronidase (3.2.1.131); α-(4-O-methyl)-glucuronidase (3.2.1.-) |
| GH30 | 1.21E-04 | 1.14E-04 | 2.65E-04 | 1.77E-04 | 2.61E-06 | 1.37E-05 | 1.66E-05 | 1.06E-04 | 6.17E-05 | 1.28 | endo-β-1,4-xylanase (EC 3.2.1.8); β-glucosidase (3.2.1.21); β-glucuronidase (EC 3.2.1.31); β-xylosidase (EC 3.2.1.37); β-fucosidase (EC 3.2.1.38); glucosylceramidase (EC 3.2.1.45); β-1,6-glucanase (EC 3.2.1.75); glucuronoarabinoxylan endo-β-1,4-xylanase (EC 3.2.1.136); endo-β-1,6-galactanase (EC:3.2.1.164); [reducing end] β-xylosidase (EC 3.2.1.-) |
| GH57 | 1.77E-04 | 1.84E-04 | 3.09E-04 | 3.57E-04 | 2.37E-05 | 4.53E-05 | 5.92E-05 | 1.47E-04 | 1.04E-04 | 1.21 | α-amylase (EC 3.2.1.1); α-galactosidase (EC 3.2.1.22); amylopullulanase (EC 3.2.1.41); cyclomaltodextrinase (EC 3.2.1.54); branching enzyme (EC 2.4.1.18); 4-α-glucanotransferase (EC 2.4.1.25) |
| GH42 | 4.56E-05 | 4.89E-05 | 7.39E-05 | 9.30E-05 | 5.92E-06 | 3.44E-05 | 1.27E-05 | 2.71E-05 | 2.76E-05 | 1.20 | β-galactosidase (EC 3.2.1.23); α-L-arabinopyranosidase (EC 3.2.1.-) |
| GH116 | 1.68E-05 | 1.46E-05 | 2.09E-05 | 3.54E-05 | 3.38E-06 | 6.71E-06 | 1.87E-05 | 6.10E-06 | 0.00E+00 | 1.20 | β-glucosidase (EC 3.2.1.21); β-xylosidase (EC 3.2.1.37); acid β-glucosidase/β-glucosylceramidase (EC 3.2.1.45); β-N-acetylglucosaminidase (EC 3.2.1.52) |
| GH77 | 1.00E-04 | 1.54E-04 | 2.42E-04 | 1.97E-04 | 1.13E-05 | 6.10E-05 | 6.12E-05 | 9.12E-05 | 1.08E-04 | 1.16 | amylomaltase or 4-α-glucanotransferase (EC 2.4.1.25) |
| GH37 | 3.33E-05 | 3.86E-05 | 7.09E-05 | 8.93E-05 | 3.83E-07 | 8.56E-06 | 8.05E-06 | 4.89E-05 | 2.43E-05 | 1.00 | α,α-trehalase (EC 3.2.1.28) |
| GH130 | 7.02E-05 | 6.18E-05 | 1.27E-04 | 1.25E-04 | 5.02E-06 | 4.21E-05 | 3.40E-05 | 5.85E-05 | 9.12E-05 | 0.96 | β-1,4-mannosylglucose phosphorylase (EC 2.4.1.281); β-1,4-mannooligosaccharide phosphorylase (EC 2.4.1.319); β-1,4-mannosyl-N-acetyl-glucosamine phosphorylase (EC 2.4.1.320); β-1,2-mannobiose phosphorylase (EC 2.4.1.-); β-1,2-oligomannan phosphorylase (EC 2.4.1.-); β-1,2-mannosidase (EC 3.2.1.-) |
| GH74 | 5.17E-05 | 1.36E-04 | 3.40E-04 | 1.21E-04 | 6.89E-05 | 3.54E-05 | 8.05E-05 | 2.75E-05 | 5.59E-05 | 0.90 | endoglucanase (EC 3.2.1.4); oligoxyloglucan reducing end-specific cellobiohydrolase (EC 3.2.1.150); xyloglucanase (EC 3.2.1.151) |
| GH94 | 6.02E-05 | 1.25E-04 | 2.30E-04 | 2.81E-04 | 4.01E-06 | 3.49E-05 | 2.18E-05 | 1.37E-04 | 1.04E-05 | 0.90 | cellobiose phosphorylase (EC 2.4.1.20); laminaribiose phosphorylase (EC 2.4.1.31); cellodextrin phosphorylase (EC 2.4.1.49); chitobiose phosphorylase (EC 2.4.1.-); cyclic β-1,2-glucan synthase (EC 2.4.1.-); cellobionic acid phosphorylase (EC 2.4.1.321); β-1,2-oligoglucan phosphorylase (EC 2.4.1.-) |
| GH120 | 1.78E-05 | 2.38E-05 | 1.43E-05 | 4.07E-05 | 1.40E-05 | 2.15E-05 | 2.43E-05 | 5.02E-06 | 4.42E-05 | 0.89 | β-xylosidase (EC 3.2.1.37) |
| GH4 | 1.09E-04 | 9.76E-05 | 1.98E-04 | 1.54E-04 | 2.93E-05 | 1.32E-04 | 9.60E-05 | 8.95E-05 | 1.36E-04 | 0.87 | maltose-6-phosphate glucosidase (EC 3.2.1.122); α-glucosidase (EC 3.2.1.20); α-galactosidase (EC 3.2.1.22); 6-phospho-β-glucosidase (EC 3.2.1.86); α-glucuronidase (EC 3.2.1.139); α-galacturonase (EC 3.2.1.67); palatinase (EC 3.2.1.-) |
| GH8 | 5.38E-05 | 7.10E-05 | 1.76E-04 | 2.21E-04 | 6.45E-06 | 9.81E-06 | 4.16E-06 | 5.89E-05 | 5.28E-05 | 0.82 | chitosanase (EC 3.2.1.132); cellulase (EC 3.2.1.4); licheninase (EC 3.2.1.73); endo-1,4-β-xylanase (EC 3.2.1.8); reducing-end-xylose releasing exo-oligoxylanase (EC 3.2.1.156) |
| GH38 | 3.70E-05 | 4.10E-05 | 6.96E-05 | 3.04E-05 | 2.89E-05 | 6.31E-05 | 8.18E-05 | 3.03E-05 | 4.33E-05 | 0.79 | α-mannosidase (EC 3.2.1.24); mannosyl-oligosaccharide α-1,2-mannosidase (EC 3.2.1.113); mannosyl-oligosaccharide α-1,3-1,6-mannosidase (EC 3.2.1.114); α-2-O-mannosylglycerate hydrolase (EC 3.2.1.170); mannosyl-oligosaccharide α-1,3-mannosidase (EC 3.2.1.-) |
| GH53 | 2.80E-05 | 2.08E-05 | 6.45E-05 | 1.05E-04 | 1.77E-06 | 1.33E-05 | 6.71E-06 | 3.29E-05 | 2.21E-05 | 0.69 | endo-β-1,4-galactanase (EC 3.2.1.89) |
| GH39 | 5.14E-05 | 4.21E-05 | 1.86E-04 | 1.19E-04 | 7.72E-06 | 2.82E-05 | 3.29E-05 | 7.36E-05 | 7.03E-05 | 0.63 | α-L-iduronidase (EC 3.2.1.76); β-xylosidase (EC 3.2.1.37) |
| GH26 | 3.95E-05 | 3.75E-05 | 1.53E-04 | 1.20E-04 | 1.22E-05 | 1.94E-05 | 2.46E-05 | 4.67E-05 | 5.25E-05 | 0.63 | β-mannanase (EC 3.2.1.78); exo-β-1,4-mannobiohydrolase (EC 3.2.1.100); β-1,3-xylanase (EC 3.2.1.32); lichenase / endo-β-1,3-1,4-glucanase (EC 3.2.1.73); mannobiose-producing exo-β-mannanase (EC 3.2.1.-) |
| GH10 | 1.42E-04 | 2.07E-04 | 5.74E-04 | 6.14E-04 | 1.41E-05 | 5.18E-05 | 5.61E-05 | 3.87E-04 | 3.09E-04 | 0.61 | endo-1,4-β-xylanase (EC 3.2.1.8); endo-1,3-β-xylanase (EC 3.2.1.32); tomatinase (EC 3.2.1.-); xylan endotransglycosylase (EC 2.4.2.-) |
| GH5 | 2.56E-04 | 2.72E-04 | 1.03E-03 | 1.07E-03 | 5.29E-05 | 1.32E-04 | 1.56E-04 | 4.23E-04 | 3.42E-04 | 0.58 | endo-β-1,4-glucanase / cellulase (EC 3.2.1.4); endo-β-1,4-xylanase (EC 3.2.1.8); β-glucosidase (EC 3.2.1.21); β-mannosidase (EC 3.2.1.25); β-glucosylceramidase (EC 3.2.1.45); glucan β-1,3-glucosidase (EC 3.2.1.58); licheninase (EC 3.2.1.73); exo-β-1,4-glucanase / cellodextrinase (EC 3.2.1.74); glucan endo-1,6-β-glucosidase (EC 3.2.1.75); mannan endo-β-1,4-mannosidase (EC 3.2.1.78); cellulose β-1,4-cellobiosidase (EC 3.2.1.91); steryl β-glucosidase (EC 3.2.1.104); endoglycoceramidase (EC 3.2.1.123); chitosanase (EC 3.2.1.132); β-primeverosidase (EC 3.2.1.149); xyloglucan-specific endo-β-1,4-glucanase (EC 3.2.1.151); endo-β-1,6-galactanase (EC 3.2.1.164); hesperidin 6-O-α-L-rhamnosyl-β-glucosidase (EC 3.2.1.168); β-1,3-mannanase (EC 3.2.1.-); arabinoxylan-specific endo-β-1,4-xylanase (EC 3.2.1.-); mannan transglycosylase (EC 2.4.1.-) |
| GH11 | 3.03E-05 | 5.94E-05 | 2.76E-04 | 3.97E-04 | 3.33E-06 | 1.24E-05 | 1.97E-05 | 1.66E-04 | 2.16E-04 | 0.29 | endo-β-1,4-xylanase (EC 3.2.1.8); endo-β-1,3-xylanase (EC 3.2.1.32) |
| GH129 | 2.64E-06 | 4.26E-06 | 2.71E-05 | 3.84E-05 | 1.22E-06 | 1.27E-05 | 7.13E-06 | 6.50E-06 | 0.00E+00 | 0.26 | α-N-acetylgalactosaminidase (EC 3.2.1.49) |
| GH113 | 2.20E-06 | 2.75E-06 | 5.48E-06 | 3.68E-05 | 1.31E-06 | 6.24E-06 | 4.16E-06 | 4.25E-06 | 1.44E-05 | 0.24 | β-mannanase (EC 3.2.1.78) |
| GH44 | 2.57E-06 | 5.03E-08 | 1.72E-05 | 2.08E-06 | 2.30E-06 | 5.26E-06 | 7.95E-07 | 2.35E-06 | 8.60E-06 | 0.24 | endoglucanase (EC 3.2.1.4); xyloglucanase (EC 3.2.1.151) |
| GH45 | 5.40E-07 | 1.18E-05 | 7.98E-05 | 1.88E-04 | 8.70E-08 | 6.87E-07 | 1.74E-07 | 1.97E-05 | 3.35E-05 | 0.13 | endoglucanase (EC 3.2.1.4) |
| GH46 | 0.00E+00 | 0.00E+00 | 1.28E-07 | 2.95E-07 | 1.21E-07 | 0.00E+00 | 3.46E-07 | 0.00E+00 | 0.00E+00 | 0.00 | chitosanase (EC 3.2.1.132) |
| GH52 | 0.00E+00 | 0.00E+00 | 1.43E-05 | 2.68E-06 | 0.00E+00 | 2.55E-07 | 1.39E-07 | 6.91E-06 | 0.00E+00 | 0.00 | β-xylosidase (EC 3.2.1.37) |
| GH54 | 0.00E+00 | 0.00E+00 | 1.19E-07 | 0.00E+00 | 1.93E-06 | 3.57E-07 | 1.29E-06 | 0.00E+00 | 0.00E+00 | 0.00 | α-L-arabinofuranosidase (EC 3.2.1.55); β-xylosidase (EC 3.2.1.37). |
| GH62 | 0.00E+00 | 0.00E+00 | 1.21E-06 | 0.00E+00 | 1.88E-06 | 1.32E-06 | 6.09E-07 | 4.54E-07 | 0.00E+00 | 0.00 | α-L-arabinofuranosidase (EC 3.2.1.55) |
| GH71 | 0.00E+00 | 0.00E+00 | 0.00E+00 | 0.00E+00 | 0.00E+00 | 3.65E-06 | 1.25E-06 | 1.51E-07 | 0.00E+00 | 0.00 | α-1,3-glucanase (EC 3.2.1.59) |
| GH75 | 0.00E+00 | 0.00E+00 | 0.00E+00 | 0.00E+00 | 1.54E-07 | 2.72E-07 | 3.99E-07 | 0.00E+00 | 0.00E+00 | 0.00 | chitosanase (EC 3.2.1.132) |
| GH79 | 0.00E+00 | 0.00E+00 | 9.32E-06 | 8.72E-06 | 0.00E+00 | 1.01E-06 | 8.73E-07 | 5.70E-07 | 6.45E-06 | 0.00 | β-glucuronidase (EC 3.2.1.31); hyaluronoglucuronidase (EC 3.2.1.36); heparanase (EC 3.2.1.166); baicalin β-glucuronidase (EC 3.2.1.167); β-4-O-methyl-glucuronidase (EC 3.2.1.-) |
| GH84 | 0.00E+00 | 0.00E+00 | 1.26E-06 | 1.22E-06 | 3.89E-07 | 0.00E+00 | 2.93E-06 | 3.50E-07 | 0.00E+00 | 0.00 | N-acetyl β-glucosaminidase (EC 3.2.1.52); hyaluronidase (EC 3.2.1.35); [protein]-3-O-(GlcNAc)-L-Ser/Thr β-N-acetylglucosaminidase (EC 3.2.1.169) |
| GH91 | 0.00E+00 | 0.00E+00 | 0.00E+00 | 3.64E-07 | 8.90E-08 | 2.63E-07 | 4.34E-07 | 0.00E+00 | 0.00E+00 | 0.00 | inulin lyase [DFA-I-forming] (EC 4.2.2.17); inulin lyase [DFA-III-forming] (EC 4.2.2.18); difructofuranose 1,2':2,3' dianhydride hydrolase [DFA-IIIase] (EC 3.2.1.-) |
